# Analysis of the peroxisome proliferator-activated receptor-β/δ (PPARβ/δ) cistrome reveals novel co-regulatory role of ATF4

**DOI:** 10.1186/1471-2164-13-665

**Published:** 2012-11-24

**Authors:** Combiz Khozoie, Michael G Borland, Bokai Zhu, Songjoon Baek, Sam John, Gordon L Hager, Yatrik M Shah, Frank J Gonzalez, Jeffrey M Peters

**Affiliations:** 1 Department of Veterinary and Biomedical Sciences and The Center for Molecular Toxicology and Carcinogenesis, The Pennsylvania State University, University Park, PA, 16802, USA; 2 Laboratory of Receptor Biology and Gene Expression, National Cancer Institute, Bethesda, MD, 20892, USA; 3 Laboratory of Metabolism, National Cancer Institute, Bethesda, MD, 20892, USA; 4 Present address: Department of Chemistry and Biochemistry, Bloomsburg University of Pennsylvania, Bloomsburg, PA, USA; 5 Present address: Genome Sciences, University of Washington, Seattle, WA, USA; 6 Present address: Department of Physiology, University of Michigan, Ann Arbor, MI, USA

**Keywords:** Peroxisome proliferator-activated receptor-β/δ, Gene expression, Keratinocytes

## Abstract

**Background:**

The present study coupled expression profiling with chromatin immunoprecipitation sequencing (ChIP-seq) to examine peroxisome proliferator-activated receptor-β/δ (PPARβ/δ)-dependent regulation of gene expression in mouse keratinocytes, a cell type that expresses PPARβ/δ in high concentration.

**Results:**

Microarray analysis elucidated eight different types of regulation that modulated PPARβ/δ-dependent gene expression of 612 genes ranging from repression or activation without an exogenous ligand, repression or activation with an exogenous ligand, or a combination of these effects. Bioinformatic analysis of ChIP-seq data demonstrated promoter occupancy of PPARβ/δ for some of these genes, and also identified the presence of other transcription factor binding sites in close proximity to PPARβ/δ bound to chromatin. For some types of regulation, ATF4 is required for ligand-dependent induction of PPARβ/δ target genes.

**Conclusions:**

PPARβ/δ regulates constitutive expression of genes in keratinocytes, thus suggesting the presence of one or more endogenous ligands. The diversity in the types of gene regulation carried out by PPARβ/δ is consistent with dynamic binding and interactions with chromatin and indicates the presence of complex regulatory networks in cells expressing high levels of this nuclear receptor such as keratinocytes. Results from these studies are the first to demonstrate that differences in DNA binding of other transcription factors can directly influence the transcriptional activity of PPARβ/δ.

## Background

Peroxisome proliferator-activated receptor-β/δ (PPARβ/δ) is a ligand activated transcription factor with particularly high abundance in small intestine, colon, liver, and skin [1-4]. In these tissues, PPARβ/δ likely has a constitutive physiological role, possibly modulated by the presence of an endogenous ligand. This is consistent with the fact that PPARβ/δ is required to mediate the induction of terminal differentiation in epithelial cells [5-10]. PPARβ/δ also attenuates inflammation and regulates glucose and lipid homeostasis [5-11]. These important physiological roles underscore the potential for targeting PPARβ/δ for the prevention and/or treatment of diverse diseases, including cancer, diabetes, metabolic syndrome, and dyslipidemias [5-10,12].

PPARβ/δ modulates cellular function by regulating gene expression through several mechanisms. For example, PPARβ/δ can interact and bind with other transcription factors, including NFκB, ERK5, and STAT3, and attenuate their signaling [5-10]. PPARβ/δ can also repress gene expression by dynamically binding to chromatin in association with co-repressors [13,14]. The most commonly described mechanism by which PPARβ/δ was thought to regulate gene expression is that binding of ligand to receptor induces a conformational change in the protein. This change in structure is accompanied by the release of co-repressors, heterodimerization with the retinoid X receptor (RXR), recruitment of co-activators and RNA polymerase II and increased transcription when this complex is bound to peroxisome proliferator response elements (PPREs) proximal to target genes. However, more recent studies indicate that nuclear receptors actually regulate gene expression through dynamic and sometimes transient interactions with chromatin rather than through static complexes occupying chromatin [15-18]. This suggests that the dynamic and transient occupancy of PPARβ/δ on chromatin must be considered when attempting to interpret receptor-DNA binding studies [15-18]. There are many different classes of enzymes that can modify nucleosome location and/or chromatin structure that will allow for nuclear receptors to dynamically and transiently bind to chromatin, and this binding can be markedly changed in response to the presence of endogenous and exogenous ligands [15-18]. Further, there is evidence that the dynamic interactions between agonist-activated nuclear receptors with their co-regulators and DNA binding sites can lead to highly variable responses including differences in: 1) the regions of chromatin occupied by the receptor, 2) expression of target genes and 3) the ultimate biological effect. Conformational changes in a receptor caused by different agonists, antagonists and partial agonists can cause differential recruitment of co-regulators resulting in alteration of the dynamics of transcriptional complexes and interactions with DNA binding sites. Thus, there are multiple levels of regulation by which PPARβ/δ can influence the expression of target genes.

Characterization of bona fide target genes directly regulated by PPARβ/δ in all tissues is incomplete. Angiopoietin-like 4 (*Angptl4*) and adipocyte differentiation-related protein (*Adrp*) are two genes that can be modulated by PPARβ/δ by direct transcriptional regulation. That *Angptl4* and *Adrp* are direct target genes is based on analyses demonstrating: 1) that functional PPREs exist proximal to these genes, and 2) confirmed promoter occupancy of PPARβ/δ following ligand activation [19-21]. In addition to these experimentally confirmed PPARβ/δ target genes, in silico screening based on genomic PPRE frequency predicted as many as 4000 to 5000 targets for PPARs in the human genome [22-24] and a comparable number of binding sites for PPARs in some cells have been confirmed by chromatin immunoprecipitation sequencing (ChIP-seq) [25]. This suggests that many PPARβ/δ binding sites and target genes remain unidentified. Moreover, recent studies have used next generation sequencing to elucidate novel regulatory roles for PPARβ/δ. For example, ChIP-seq was used to demonstrate that PPARβ/δ and PPARγ can be exchanged on target gene in adipocytes, following ligand activation of PPARγ [26]. Thus, the present studies were designed to examine novel regulation of PPARβ/δ-dependent gene transcription in keratinocytes.

## Results

### PPARβ/δ-dependent regulation of genes in keratinocytes

**Figure 1:**
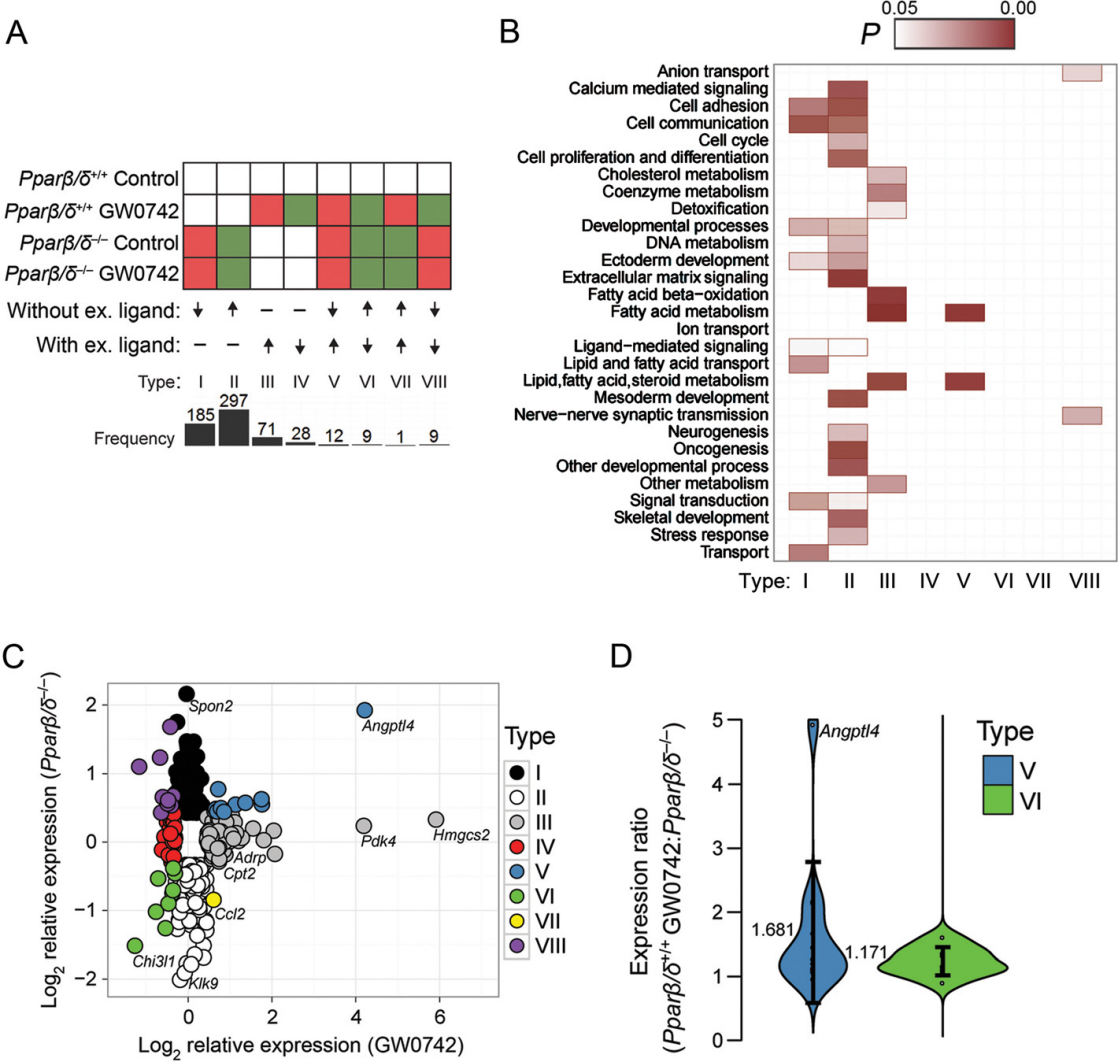
**Eight distinctly different PPARβ/δ-dependent mechanisms of transcriptional regulation. **(**A**) 612 genes were categorized into eight different response types. Relative expression was based by comparison with control, wild-type mouse keratinocytes. White indicates basal expression; red indicates higher expression compared to control, wild-type mouse keratinocytes; green indicates lower expression compared to control, wild-type mouse keratinocytes. An up arrow indicates repression and a down arrow indicates activation. The number of genes within each category is shown in the bar graph. (**B**) Enrichment of functional gene categories of the 612 differentially regulated genes within each of the response types. Statistically significant enriched gene categories were identified by PANTHER (*P* ≤ 0.05) and are indicated by a box, with darker shaded boxes depicting lower *P* values. (**C**) Comparison of gene expression profiles observed with disruption of PPARβ/δ (y axis) and activation with GW0742 in wild-type keratinocytes (x axis) for the 612 genes as categorized by response type. Relative gene expression levels were log transformed and a ratio relative to control wild-type keratinocytes was plotted for each gene. (**D**) Violin plots of the log transformed ratios of gene expression in wild-type keratinocytes treated with GW0742 compared to *Pparβ/δ*^−/−^ keratinocytes for the type V and VI responses. Each data point represents the mean of at least three independent biological replicates. The mean ratio is shown next to each violin plot.

**Figure 2:**
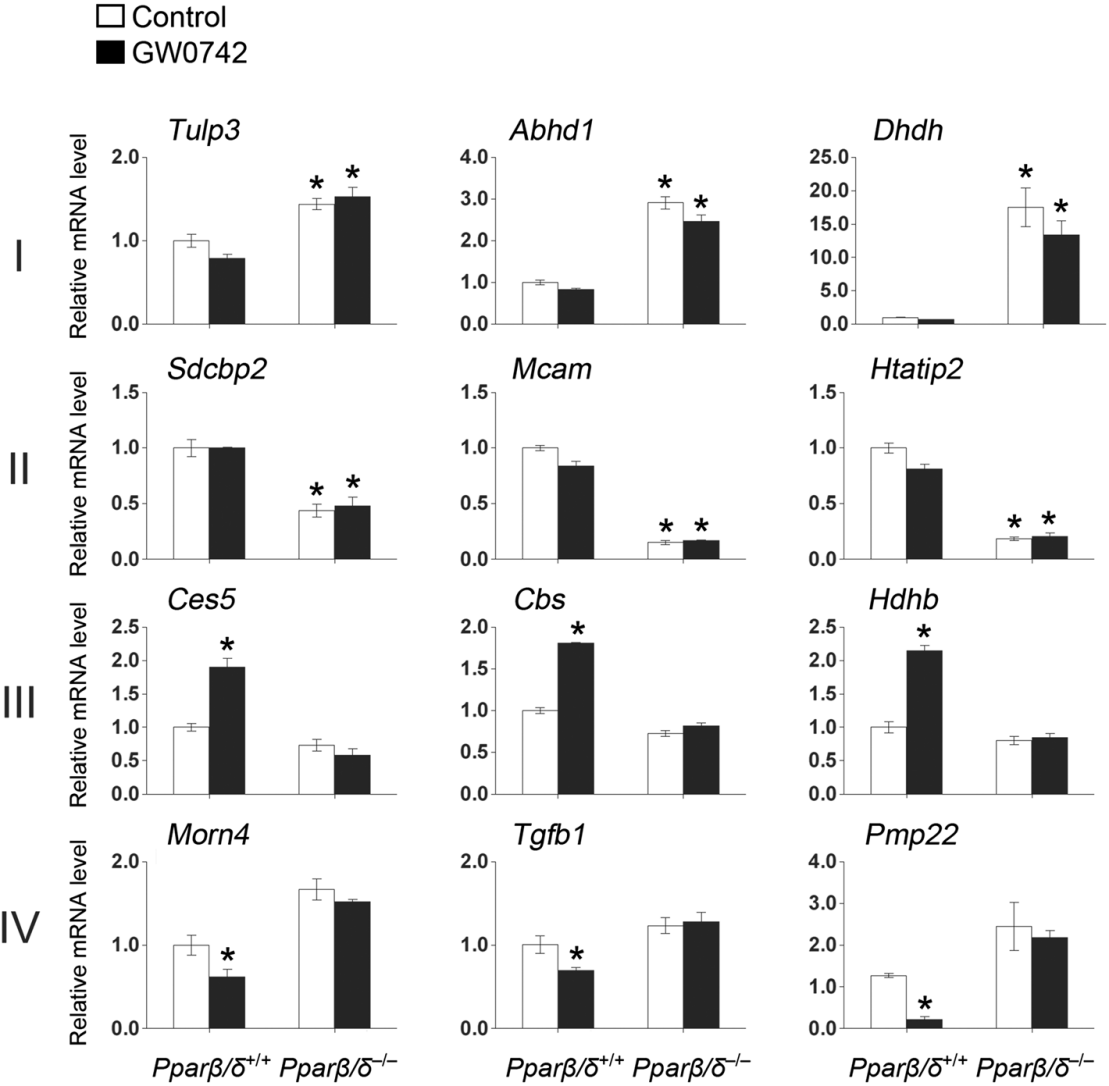
**qPCR confirmation of PPARβ/δ-dependent changes in gene expression detected by microarray analysis. **(**A**) qPCR analysis of the four most common types of regulation (I-IV) was examined using RNA from control and GW0742-treated wild-type (*Pparβ/δ*^+/+^) and *Pparβ/δ*-null (*Pparβ/δ*^−/−^) keratinocytes. *Significantly different than control, *P* ≤ 0.05 as determined by ANOVA and post-hoc testing.

Six hundred and twelve genes were identified by genome-wide expression profiling that were differentially regulated by either ligand, disruption of PPARβ/δ, or both (Figure 1A, Table 1, Additional file 1: Table S2). These genes were categorized into four major response types: (I) repression without exogenous ligand (n=185), (II) activation without exogenous ligand (n=297), (III) activation with exogenous ligand (n=71), and (IV) repression with exogenous ligand (n=28). qPCR confirmed these four common types of PPARβ/δ-dependent changes in gene expression detected by microarray analysis (Figure 2). Combined, these comprised 94.9% of the genes identified that were differentially regulated by either ligand, disruption of PPARβ/δ, or both. An additional four response types exhibiting combined responses were also observed: (V) repression without exogenous ligand and activation with exogenous ligand (n=12), (VI) activation without exogenous ligand and repression with exogenous ligand (n=9), (VII) activation with and without exogenous ligand (n=1), and (VIII) repression with and without exogenous ligand (n=9) (Figure 1A, Table 1). The latter four response types only comprised ~5% of the genes modulated by GW0742 and/or disruption of PPARβ/δ.

**Table 1:** Types of transcriptional responses observed following ligand activation of PPARβ/δ in mouse primary keratinocytes

**Type**	**Without exogenous ligand**	**With exogenous ligand**
I	Repression	–
II	Activation	–
III	–	Activation
IV	–	Repression
V	Repression	Activation
VI	Activation	Repression
VII	Activation	Activation
VIII	Repression	Repression

The effect without exogenous ligand reflects the response in expression of a particular gene observed in control *Pparβ/δ*-null keratinocytes as compared to control wild-type keratinocytes. For this column, repression indicates that gene expression is higher in *Pparβ/δ*-null cells as compared to wild-type cells and activation indicates that gene expression is lower in *Pparβ/δ*-null cells as compared to wild-type cells. The effect with exogenous ligand reflects the response in expression of a particular gene observed in wild-type keratinocytes treated with the highly specific PPARβ/δ ligand GW0742 as compared to control wild-type keratinocytes. For this column, activation indicates that gene expression is higher in GW0742-treated wild-type cells as compared to control wild-type cells and repression indicates that gene expression is lower in GW0742-treated wild-type cells as compared to control wild-type cells.

Characterization of the PPARβ/δ target genes and their transcriptional responses was undertaken by functional category enrichment analysis [27,28]. 50% of the enriched functional categories were common between type I and type II responses (Figure 1B). Genes that regulate fatty acid metabolism were common between type III and type V responses (Figure 1B). The level of gene expression observed following ligand activation was compared to changes in gene expression observed by disruption of PPARβ/δ (Figure 1C). This analysis revealed clustering for the eight different response types, but there were differences in the magnitude of change found for each of the response types (Figure 1C). The conventional view accounting for combined responses involving activation/repression in both the presence and absence of exogenous ligand (types V and VI, e.g. *Lpcat3*) suggests that a ligand-mediated switch occurs between repression and activation (type V) or vice-versa (type VI) [13]. For example, in the absence of PPARβ/δ, expression of *Lpcat3* is enhanced because PPARβ/δ represses expression, whereas ligand activation of PPARβ/δ increases expression of *Lpcat3*. This phenomenon was also observed with type VI genes, consistent with a ligand-mediated release of an activating complex. However, it is important to point out that the level of ligand-dependent expression typically exhibited in type V genes was of generally greater magnitude (average 1.6 fold) as compared to the level of expression observed when PPARβ/δ was disrupted, but this effect was not found for type VI responses (Figure 1D). This suggests that ligand and receptor-dependent regulation of gene expression can be mediated by distinctly different mechanisms, even for a single gene.

### Genome-wide characterization of PPARβ/δ occupancy on chromatin

**Figure 3:**
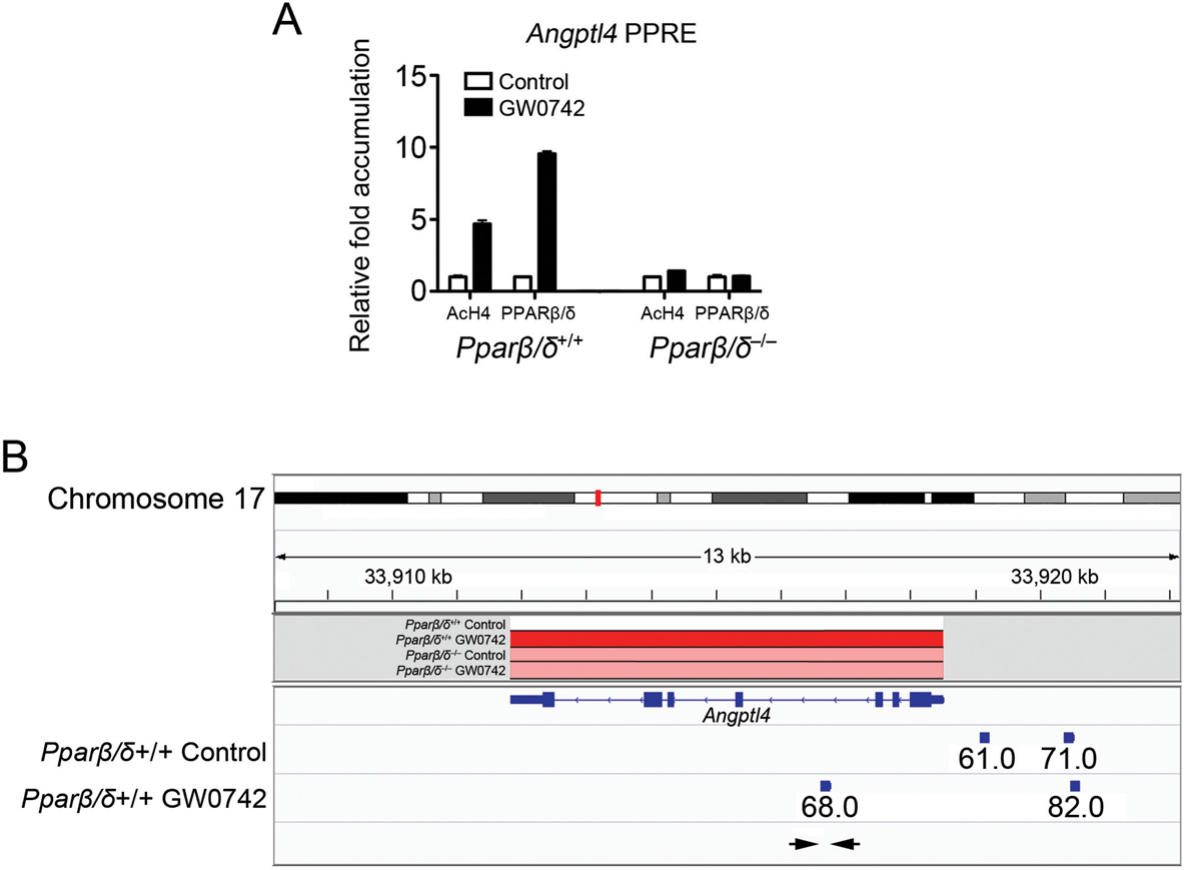
**Characterization of ChIP-grade anti-PPARβ/δ antibody. **(**A**) ChIP analysis for AcH4 or PPARβ/δ occupancy on chromatin from wild-type or *Pparβ/δ*-null keratinocytes treated with or without GW0742 (0.2 μM). qPCR was performed using chromatin immunoprecipitated with either an anti-AcH4 or the 8099 anti-PPARβ/δ antibody. (**B**) Integrated overview of microarray expression and ChIP-seq data for Angptl4. The region of the *Angptl4* gene is shown in red on chromosome 17, and the corresponding genomic location of this gene is depicted below. Relative expression of *Angptl4* is shown for the four treatment groups (wild-type and *Pparβ/δ*-null, with and without GW0742) with white representing basal expression, red representing greatly enhanced expression and pink representing enhanced expression. The exonic and intronic organization of the *Angptl4* gene is depicted with boxes (exons) and lines (introns). Regions associated with increased PPARβ/δ occupancy based on ChiP-seq analysis (peaks) are shown in boxes corresponding to the genomic regions depicted above with the Z score listed below. Note the intronic region used for ChIP in (A), which is highlighted by arrows, is associated with a peak detected by ChIP-seq.

ChIP-seq was performed to examine the molecular mechanisms by which PPARβ/δ regulates gene expression. To accomplish this, a ChIP-grade antibody is required. A polyclonal anti-PPARβ/δ antibody [2] was ~94X more efficient for immunoprecipitation of vitro translated PPARβ/δ as compared to one commercially available anti-PPARβ/δ antibody [2]. Further, this antibody was effective for demonstrating increased promoter occupancy of PPARβ/δ on the *Angptl4* promoter in mouse keratinocytes following ligand activation of PPARβ/δ (Figure 3A) and was used for ChIP-seq to identify PPARβ/δ cistromes in keratinocytes. Between 17,575,718 and 27,509,922 reads per sample were obtained by ChIP-seq and more than 98% of these reads were retained after quality control. Of these reads, between 66 and 73% were successfully mapped to the mouse genome for the control and GW0742-treated samples (Table 2).

**Table 2:** **ChIP-seq reads of DNA from wild-type (*Pparβ/δ***^***+/+***^**) and *Pparβ/δ*-null (*Pparβ/δ***^**−/−**^**) keratinocytes**

	***Pparβ/δ***^***+/+***^			***Pparβ/δ***^**−/−**^		
	**Input**	**DMSO**	**GW0742**	**Input**	**DMSO**	**GW0742**
Reads (Run 1)	6173615	11774655	17489428	13397018	9111547	10955382
Reads (Run 2)	11402103	9298650	10020494	13963586	11731976	11870020
Total Reads	17575718	21073305	27509922	27360604	20843523	22825402
Quality Control Passed Reads	17521514	20910231	27379614	27247515	20625001	22363483
% QC Passed	99.69	99.23	99.53	99.59	98.95	97.98
Mapped Reads	16016425	15351905	19552539	25239456	14630180	14770720
% Mapped	91.41	73.42	71.41	92.63	70.93	66.05

The number of reads per sample is shown for each of two runs, along with the total number of reads obtained for bioinformatic analysis. The percentage of total reads that passed quality control as described in Materials and Methods (% QC Passed) and the number (Mapped Reads) and percentage of reads that were successfully mapped to the mouse genome (% Mapped) are shown.

**Figure 4:**
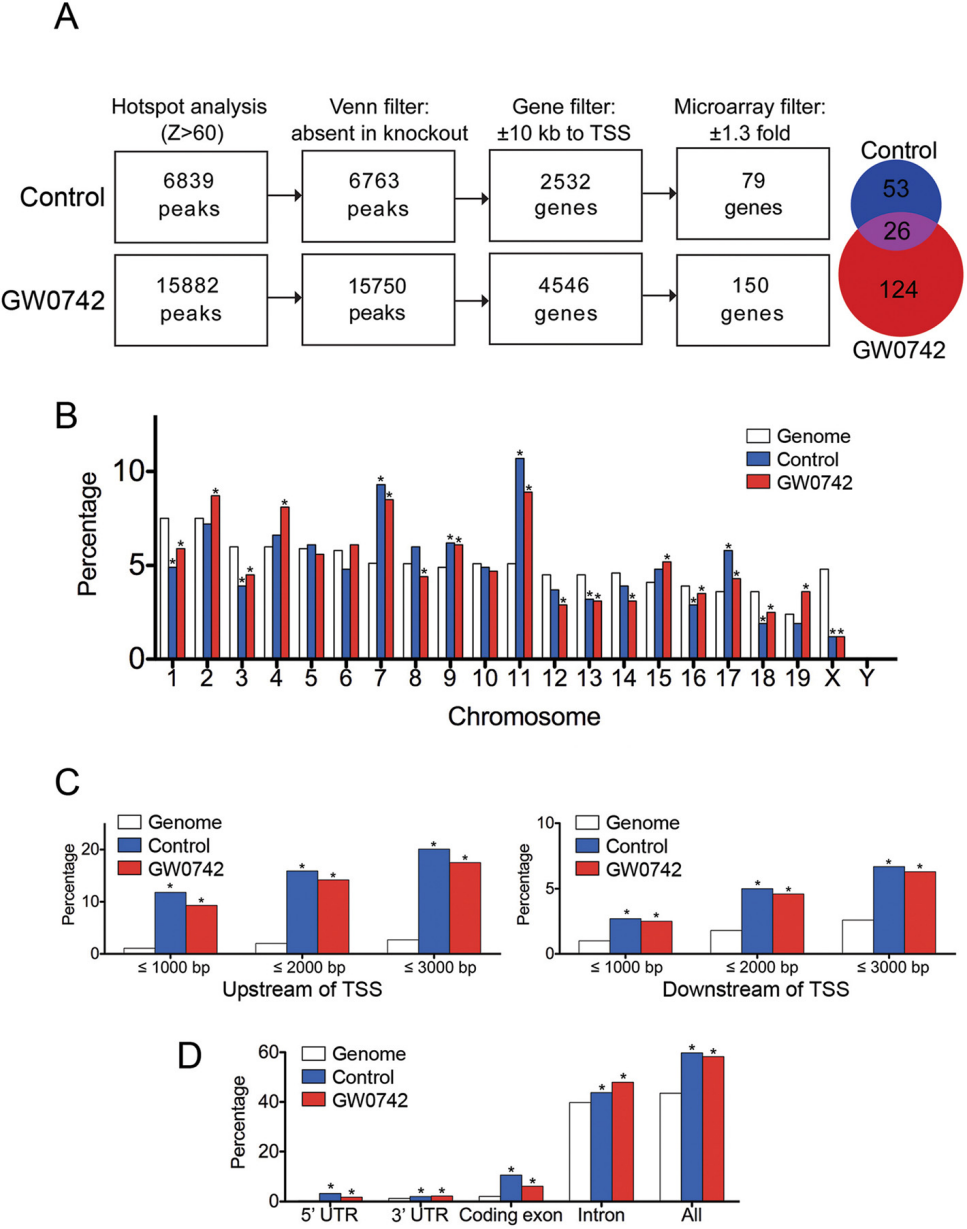
**Characterization of PPARβ/δ binding on chromatin in keratinocytes. **(**A**) ChIP-seq analysis was performed to identify regions of chromatin with PPARβ/δ occupancy (Hotspot analysis). These data were filtered to correct for background by removing peaks detected in *Pparβ/δ*-null samples and to identify peaks within a ± 10 kb region of any transcription start site (TSS). These filtered data were then compared with expression levels of genes to identify direct PPARβ/δ target genes that were regulated with and without exogenous ligand (**B**) Annotation of ChIP-seq peaks with chromosome localization. The percentage of the mouse genome on each chromosome is shown in relationship to the percentage of the ChIP-seq peaks detected that were localized to each chromosome. (**C**) Annotation of ChIP-seq peaks either upstream or downstream from TSS. The percentage of the ChIP-seq peaks detected that were localized between 1000 and 3000 bp from the TSS is shown as compared to the percentage of the mouse genome in these regions. (**D**) Annotation of ChIP-seq peaks in the intragenic 5′-UTR, 3′-UTR, coding exons and introns. The percentage of the ChIP-seq peaks detected in intragenic 5′-UTR, 3′-UTR, coding exons and introns is shown as compared to the percentage of the mouse genome in these regions. *Significantly different from respective genomic control, *P* ≤ 0.05 as determined by ANOVA and post-hoc testing.

Bioinformatic analysis of ChIP-seq data revealed occupancy of PPARβ/δ on 6,839 sites in chromatin from control cells (binding without exogenous ligand) and 15,882 sites in chromatin from GW0742 treated cells (binding with exogenous ligand); their absence in equivalent *Pparβ/δ*-null cells confirmed specificity (Figure 4A). The region of DNA amplified by ChIP qPCR showing increased promoter occupancy of PPARβ/δ on the *Angptl4* gene following ligand activation (Figure 3A) was also associated with significant occupancy of PPARβ/δ based on ChIP-seq analysis (Figure 3B) confirming specificity of promoter occupancy detected by ChIP-seq. Annotation of the genomic features associated with the identified peaks revealed unique patterns of PPARβ/δ occupancy on specific chromosomes (Figure 3B). As compared to the percentage of bases in the entire mouse genome per chromosome, the percentage of peaks identified by ChIP-seq that were associated with PPARβ/δ binding was significantly greater on chromosomes 7, 9, 11 and 17 and significantly less on chromosomes 1, 3, 13, 18 and X in control, wild-type keratinocytes (Figure 4B). As compared to the percentage of bases in the entire mouse genome per chromosome, the percentage of peaks identified by ChIP-seq that were associated with PPARβ/δ binding was significantly greater on chromosomes 2, 4, 15 and 19 and significantly less on chromosomes 8, 12 and 14 following ligand activation of PPARβ/δ (Figure 4B). Annotation of the genomic features associated with the identified peaks also revealed that the majority of PPARβ/δ binding to chromatin is found at intronic sites (43.8 – 47.9%), with significant enrichment also observed in the upstream (9.3 – 20.1%) and downstream (2.5 – 6.7%) regions from the TSS; which varied depending on the distance upstream from the transcriptional start site (TSS); Figures 4C, 4D). However, as compared to the percentage of bases in the entire mouse genome that represents intronic sequences (39.8%), the percentage of peaks identified by ChIP-seq that were associated with PPARβ/δ binding (43.8 – 47.9%) was not strikingly different (Figure 4D). In contrast, as compared to the percentage of bases in the entire mouse genome that are in relatively close proximity to the TSS (≤ 2.7%), the percentage of peaks identified by ChIP-seq that were associated with PPARβ/δ binding (9.3 – 20.1%) was between 3- and 7-fold higher (Figure 4C).

### Integrative analysis of the PPARβ/δ transcriptome and cistrome

**Figure 5:**
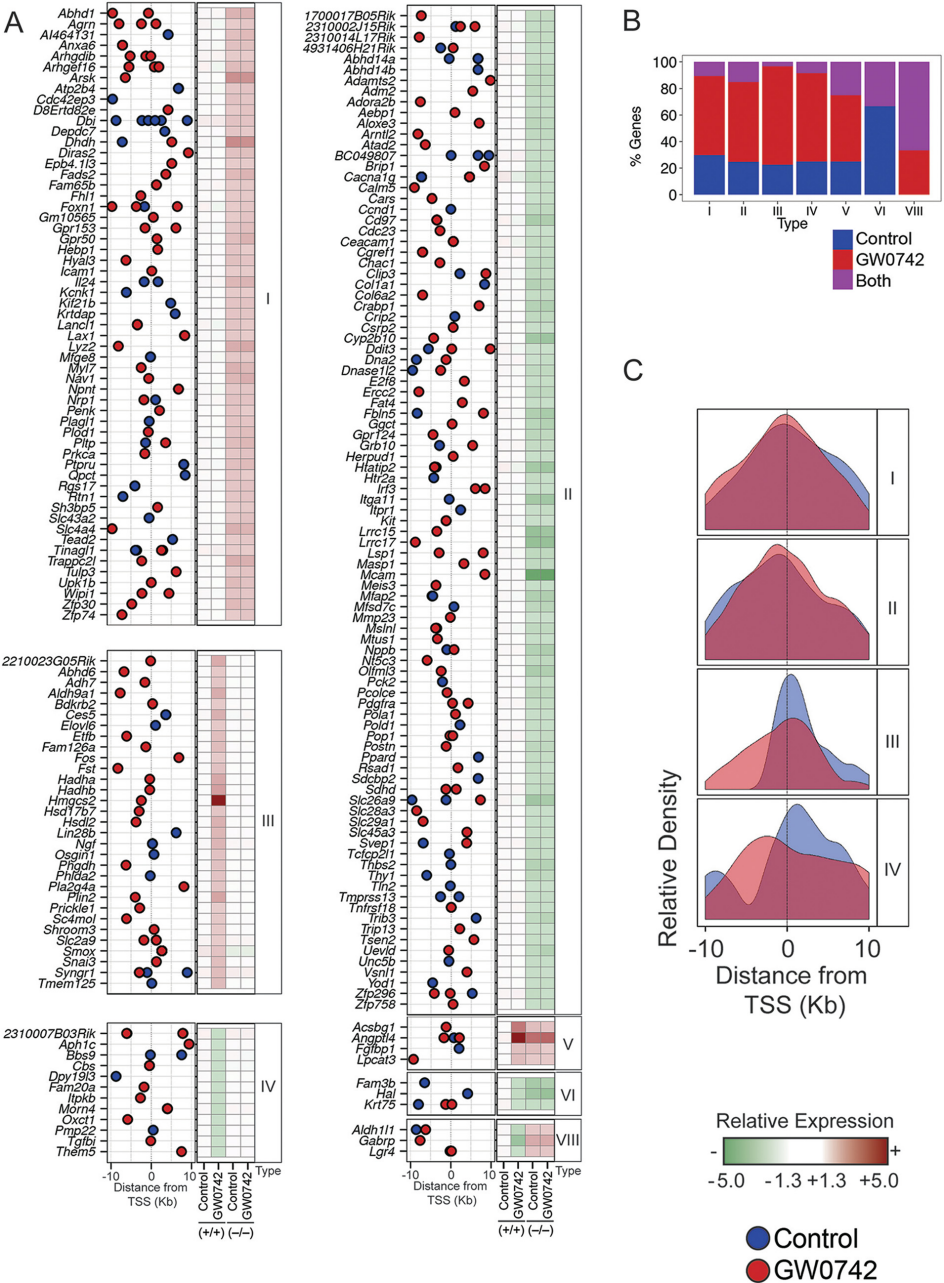
**Characterization of different response types by integration of cistromic and genome-wide expression profiling analysis. **(**A**) Comparison of PPARβ/δ occupancy of the 203 direct target genes between the different response types. PPARβ/δ occupancy is shown without (blue dots) and with (red dots) exogenous ligand (GW0742) in relation to the TSS. Relative expression of the target genes in wild-type (+/+) and *Pparβ/δ*-null (−/−) samples is shown in the heat map to the right of this analysis. (**B**) Relative distribution of PPARβ/δ occupancy in the absence or presence of exogenous ligand for the different response types. (**C**) The distribution of PPARβ/δ occupancy in relation to the TSS for the direct target genes for the four major response types.

Binding regions were assigned gene names according to the nearest TSS. As transcription factors may regulate genes at distal loci (e.g. > 30 kb), a high-confidence binding site and direct target gene set was obtained by identifying genes exhibiting both PPARβ/δ occupancy within ± 10 kB of the gene, and PPARβ/δ-dependent differential expression as revealed by microarray analysis (Figures 4A, 5A). 79 genes were identified that exhibited PPARβ/δ occupancy on chromatin without exogenous ligand and exhibited PPARβ/δ-dependent differential expression (Figure 4A). Further, 150 genes were identified that exhibited PPARβ/δ occupancy on chromatin with exogenous ligand and exhibited PPARβ/δ-dependent differential expression (Figure 4A). Twenty-six of these genes exhibited PPARβ/δ occupancy on chromatin with and without exogenous ligand and exhibited PPARβ/δ-dependent differential expression (Figure 4A). This integrated, high-confidence “direct target gene set” and “binding region set” was utilized for analyses of PPARβ/δ-chromatin interactions (Figure 5).

**Figure 6:**
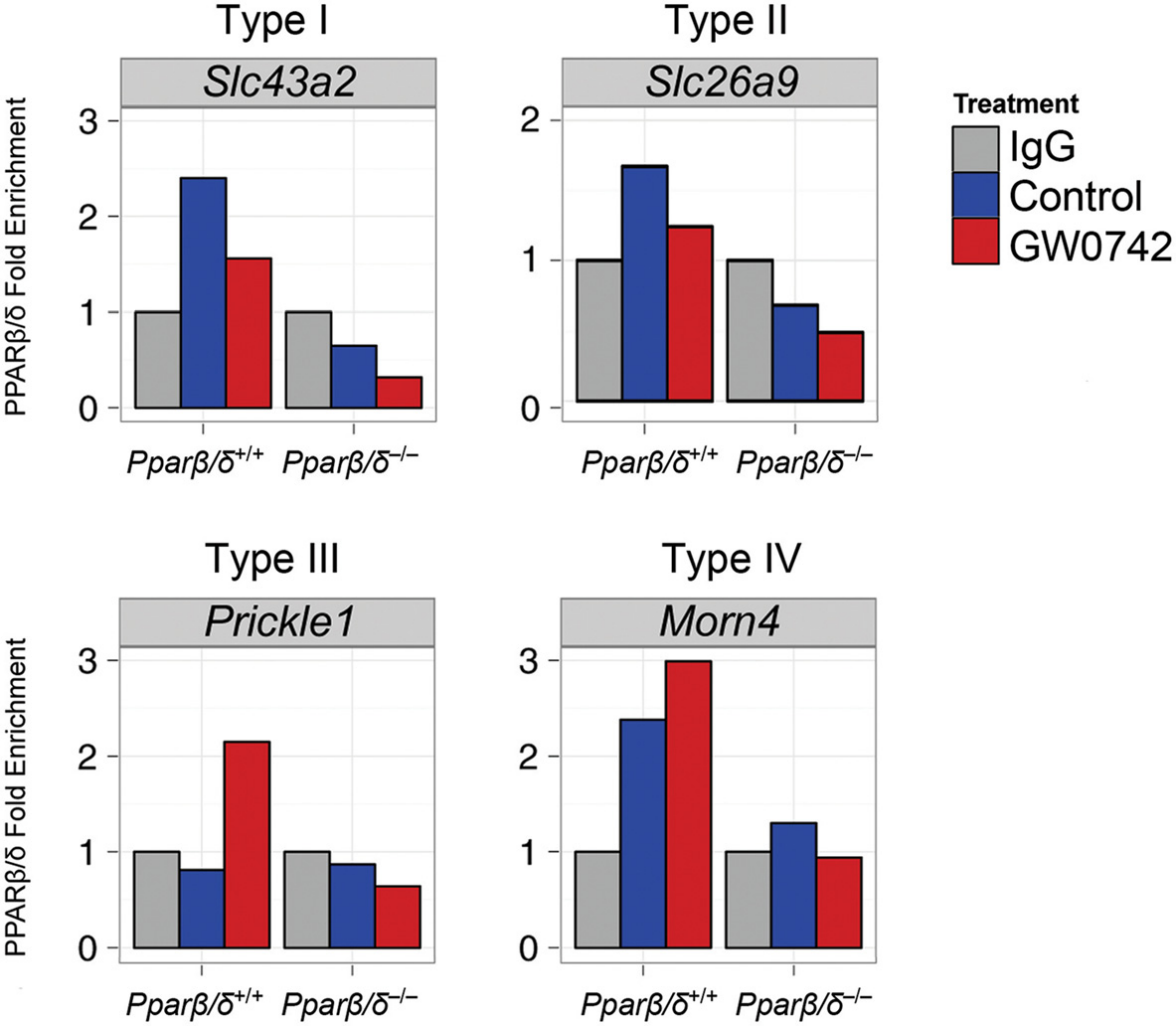
**ChiP-qPCR validation of PPARβ/δ occupancy for the four major response types.** Chromatin from keratinocytes was used for ChIP and the immunoprecipitated chromatin used for qPCR of regions where PPARβ/δ occupancy was detected by ChIP-seq for one representative gene of each major response type.

Of the 612 PPARβ/δ-dependent differentially regulated genes detected with the microarray analysis, 203 (33%) of these genes also displayed PPARβ/δ occupancy within 10 kb of the TSS (Additional file 2: Table S3). Fifty-seven of the 203 direct target genes exhibited a type I response and promoter occupancy of PPARβ/δ was found in 23 (40%) of these genes within 10 kb of the TSS in the absence of exogenous ligand (Figures 5A, 5B). Interestingly, in 34 of these genes, PPARβ/δ occupancy was only detected in the presence of exogenous ligand (Figures 5A, 5B). Of the 93 direct PPARβ/δ genes exhibiting a type II response, promoter occupancy of PPARβ/δ was found in 37 (40%) of these genes within 10 kb of the TSS in the absence of exogenous ligand (Figures 5A, 5B) but in 56 of these genes, PPARβ/δ occupancy was only detected in the presence of exogenous ligand (Figures 5A, 5B). The occupancy of PPARβ/δ within 10 kb of the TSS found with and without exogenous ligand was relatively equally distributed with more predominant occupancy near the TSS for both type I and type II response genes (Figure 5C). Thirty-one of the 203 direct target genes exhibited a type III response and promoter occupancy of PPARβ/δ within 10 kb of the TSS was found for all of these genes but was found in the absence and/or presence of exogenous ligand (Figures 5A, 5B). For type III response genes, occupancy of PPARβ/δ without exogenous ligand occurred more predominantly near the TSS with some skewness in the region downstream of the TSS (Figure 5C). In the presence of exogenous ligand, the occupancy of PPARβ/δ observed for type III response genes was predominant near the TSS but there was a shift in binding towards the region upstream of the TSS compared to the occupancy of PPARβ/δ observed without exogenous ligand (Figure 5C). Of the 12 direct PPARβ/δ genes that exhibited a type IV response, promoter occupancy of PPARβ/δ within 10 kb of the TSS was found for all of these genes in the absence and/or presence of exogenous ligand (Figures 5A, 5B). For type IV response genes, in the absence of exogenous ligand occupancy of PPARβ/δ was near the TSS with a cluster of binding in the region upstream of the TSS and some skewness in the region downstream of the TSS (Figure 4C). The occupancy of PPARβ/δ observed for type IV response genes in the presence of exogenous ligand was shifted towards the region upstream of the TSS compared to the occupancy of PPARβ/δ observed without exogenous ligand (Figure 5C). Four of the 203 direct target genes exhibited a type V response and promoter occupancy of PPARβ/δ within 10 kb of the TSS was found for all of these genes in the absence and/or presence of exogenous ligand (Figures 5A, 5B). Of the 3 direct PPARβ/δ genes that exhibited a type VI response, promoter occupancy of PPARβ/δ within 10 kb of the TSS was found for all of these genes in the absence and/or presence of exogenous ligand (Figures 5A, 5B). The single type VII response gene detected by microarray analysis did not exhibit occupancy of PPARβ/δ based on this analysis. Three of the 203 direct target genes exhibited a type VIII response and promoter occupancy of PPARβ/δ within 10 kb of the TSS was found for all of these genes in the absence and/or presence of exogenous ligand (Figures 5A, 5B). ChIP-qPCR was performed for representative genes from the four major response types to confirm PPARβ/δ occupancy and chromatin modifications (Figure 6).

### Unique DNA binding motifs near sites of PPARβ/δ occupancy on chromatin

**Figure 7:**
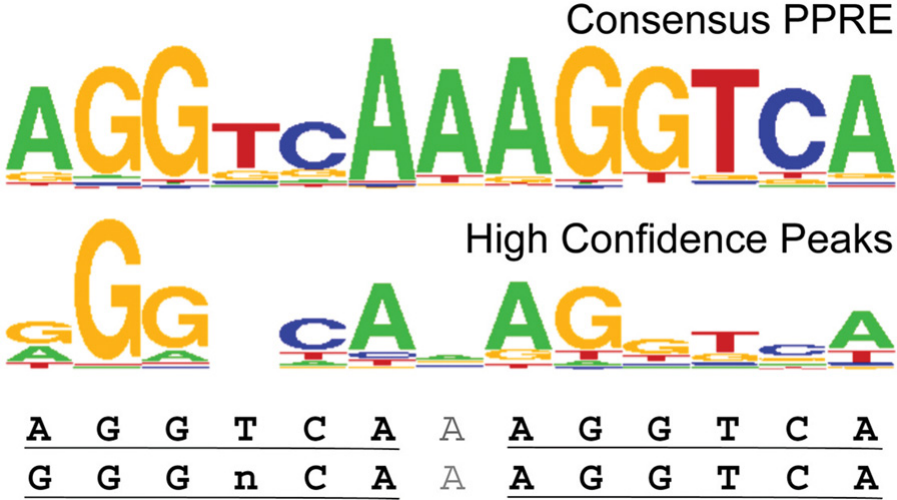
**Consensus PPRE sequence identified by binding site analysis.** A consensus PPRE motif for PPARβ/δ was determined using a list of 53 validated PPRE consensus sequences obtained from the literature. These data were used for comparison with DNA sequences where PPARβ/δ occupancy was detected by ChIP-seq to reveal the most consistent PPRE motif.

**Figure 8:**
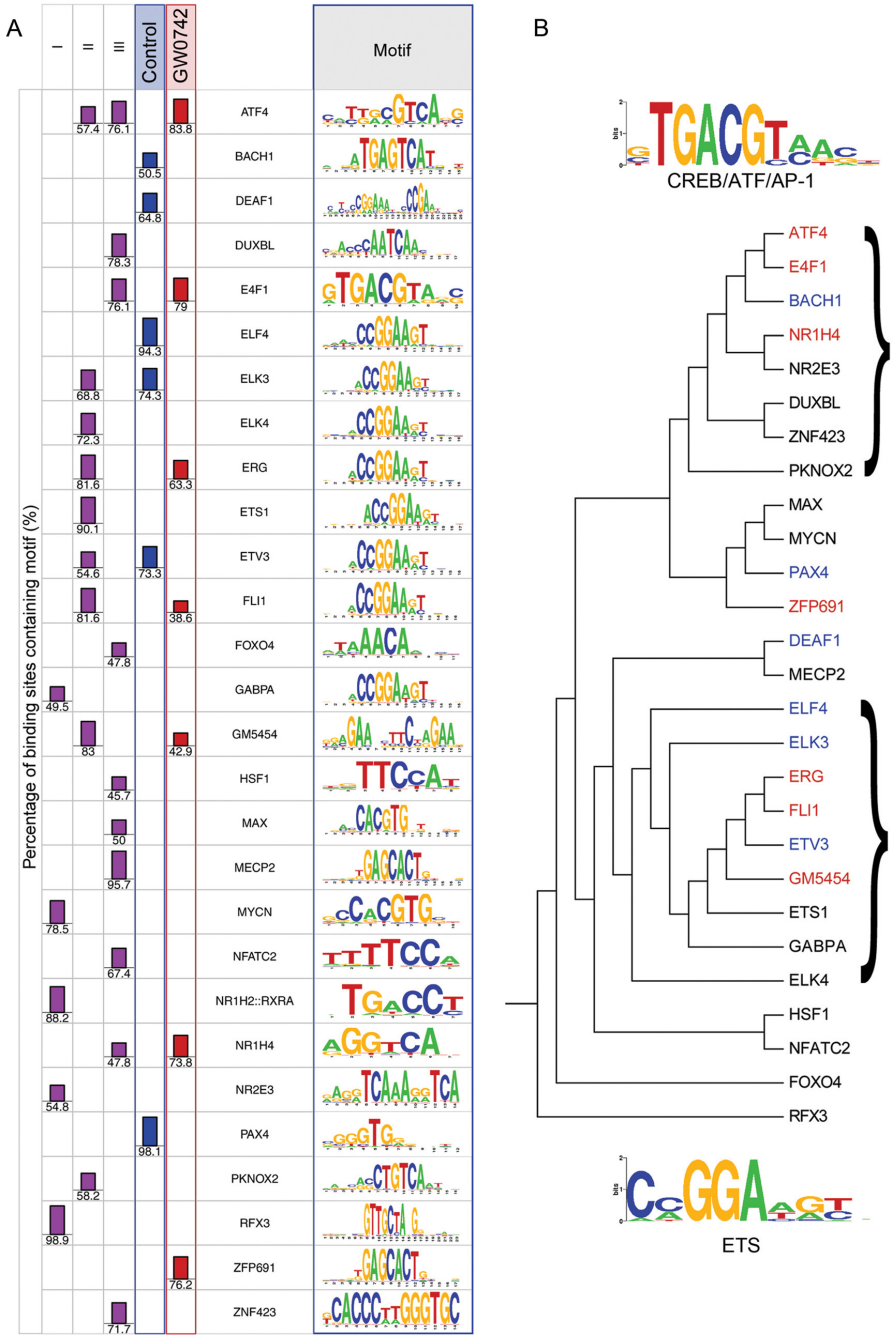
**Transcription factor DNA binding motifs proximal to PPARβ/δ binding regions. **(**A**) Enrichment of transcription factor binding motifs proximal to PPARβ/δ binding regions observed in the absence (DMSO) or presence (GW0742) of exogenous ligand, or with the different response types (I-III) are shown. Values represent the percentage of binding regions containing the indicated transcription factor DNA binding motif. The indicated consensus transcription factor DNA binding motifs are also shown. (**B**) Sequence similarities between the transcription factor DNA binding motifs were identified by multiple sequence alignments and are shown in a phylogenetic tree. The parentheses indicate the two most common families observed (CREB/ATF/AP1 and ETS).

A consensus PPRE motif for PPARβ/δ was generated using a list of 53 validated PPRE consensus sequences obtained from the literature [29] using STAMP [30] (Figure 7). This sequence was used to search for matches in the ChIP-seq peaks in both control and GW0742-treated samples. The consensus PPRE motif identified from this analysis was GGGNCAAAGGTCA (Figure 7). The RXR half-site was conserved whereas the PPARβ/δ half-site showed some variation as compared to the consensus PPRE motif based on the published literature (Figure 7). Consensus binding sites for a number of other transcription factors were also found in proximity to regions where PPARβ/δ occupancy was noted (Figure 8A). For example, in control chromatin from wild-type keratinocytes not treated with an exogenous ligand, a high percentage (73.3 – 98.1%) of consensus binding sites for ELF4, ELK3, ETV3 and PAX4 were also found in ChIP-seq peaks (Figure 8A). Additionally, in chromatin from wild-type keratinocytes treated with GW0742, a high percentage (63.3 – 83.8%) of consensus binding sites for ATF4, E4F1, ERG, NR1H4, and ZFP691 were also found in ChIP-seq peaks (Figure 8A). Differences in the presence of consensus binding sites for a number of transcription factors were also found in regions where PPARβ/δ occupancy was noted between response types (Figure 8A). For genes exhibiting a type I response, a high percentage (78.5 – 98.9%) of consensus binding sites for MYCN, NR1H2:RXR, and RFX3 were also found in ChIP-seq peaks in chromatin from wild-type keratinocytes (Figure 8A). A high percentage (68.8 – 90.1%) of consensus binding sites for ELK3, ELK4, ERG, TS1, FLI1, and GM5454 were also found in ChIP-seq peaks in chromatin from wild-type keratinocytes for genes exhibiting a type II response (Figure 8A). For genes exhibiting a type III response, a high percentage (71.7 – 95.7%) of consensus binding sites for ATF4, DUXBL, E4F1, MECP2, and ZNF423 were also found in ChIP-seq peaks in chromatin from wild-type keratinocytes (Figure 8A). Related transcription factors were grouped by phylogenic analysis revealing two major families of transcription factor binding sites, ETS and CREB/ATF/AP1, that were associated near regions where PPARβ/δ was found to occupy (Figure 8B). Interestingly, binding sites for the ETS family of transcription factors were found near regions of chromatin occupied by PPARβ/δ in either the presence or absence of exogenous ligand, whereas binding sites for the CREB/ATF/AP1 family of transcription factors were more commonly found in regions of chromatin occupied by PPARβ/δ only in the presence of ligand (Figure 8B). No significant enrichment of consensus binding sites for other transcription factors were found in ChIP-seq peaks from the type IV-VIII response genes.

### Co-regulation of PPARβ/δ target genes by ATF4

**Figure 9:**
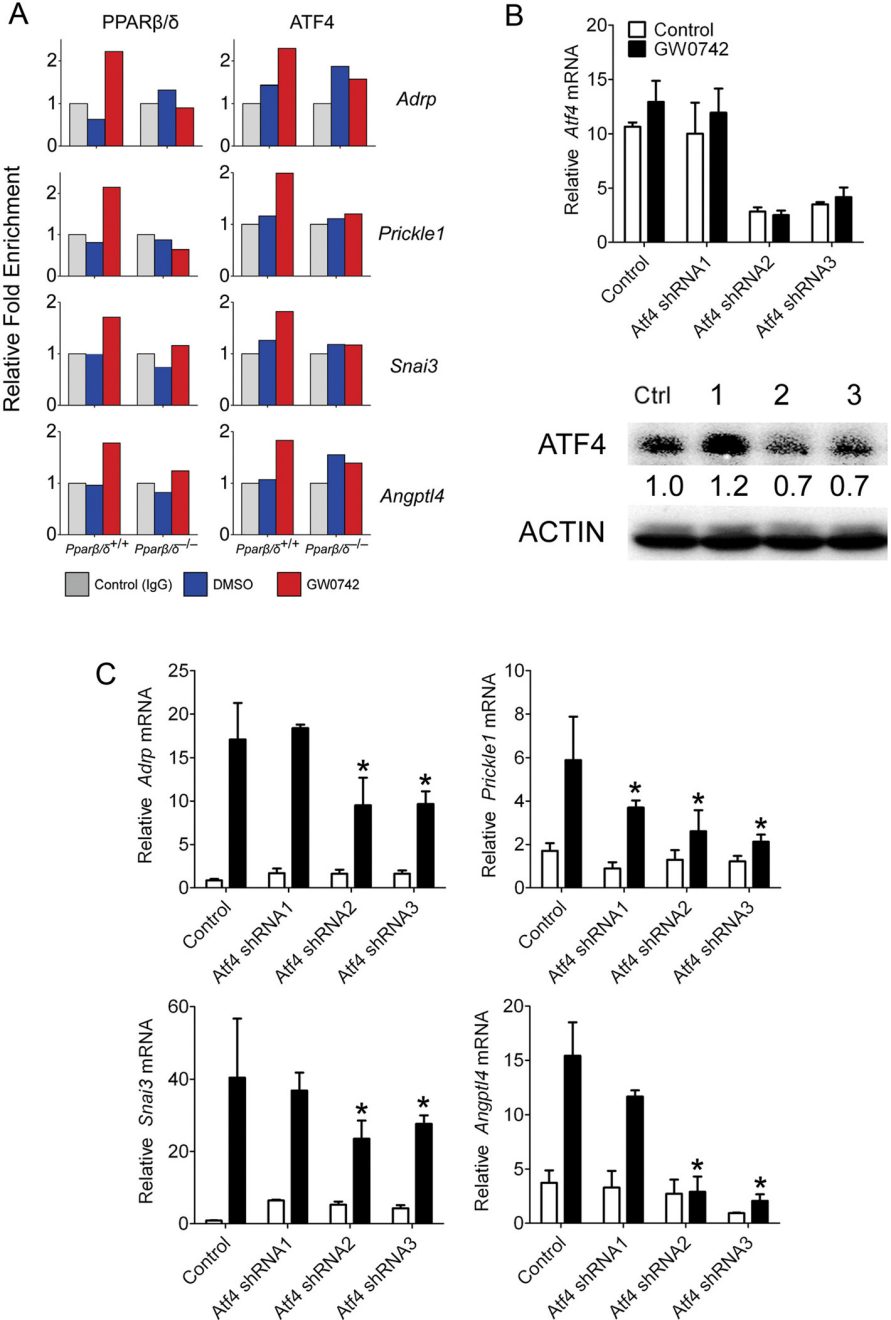
**PPARβ/δ and ATF4 co-operatively modulate gene expression. **(**A**) ChIP assays demonstrating promoter occupancy of both PPARβ/δ and ATF4 on representative type III target genes (*Adrp*, *Prickle1* and *Snai3*) and one type V target gene (*Angptl4*). (**B**) Knockdown of ATF4 in SP1 cells. Upper panel shows mRNA analysis demonstrating effective knockdown of ATF4 by two of three shRNA vectors compared to control. Lower panel shows western blot analysis confirming ATF4 knockdown. (**C**) Effect of ATF4 knockdown on ligand activation of PPARβ/δ in SP1 cells. qPCR analysis of representative type III target genes (*Adrp*, *Prickle1* and *Snai3*) and one type V target gene (*Angptl4*). Values represent the mean ± S.E.M. *Significantly lower ligand induction as compared to control as determined by ANOVA and post-hoc testing.

Because ATF4 binding motifs were commonly observed proximal to PPARβ/δ binding regions, ChIP assays were performed to confirm that both transcription factors occupied these regions of DNA. Interestingly, for three type III response genes (*Adrp*, *Prickle1* and *Snai3*) and one type V gene (*Angptl4*), ligand activation of PPARβ/δ was associated with an increase in promoter occupancy of both PPARβ/δ and ATF4 (Figure 9A). This effect was not observed in *Pparβ/δ*-null cells. Because effective knockdown of ATF4 could not be achieved in primary keratinocytes, the SP1 keratinocyte cell line [31] was used to examine the effect of ATF4 knockdown. Of the three ATF4 shRNA vectors used, effective knockdown of ATF4 was achieved with two of the vectors (Figure 9B). Interestingly, when ATF4 was knocked down, the relative increase in gene expression of the type III genes *Adrp*, *Prickle1*, and *Snai3* observed following ligand activation of PPARβ/δ was reduced (Figure 9C). Additionally, the relative increase in gene expression of the type V gene *Angptl4* observed following ligand activation of PPARβ/δ was also markedly reduced when ATF4 was knocked down (Figure 9C).

## Discussion

Results from the present study are the first to demonstrate genome-wide changes in gene expression mediated specifically by PPARβ/δ in mouse keratinocytes. Keratinocytes provide an outstanding model to examine gene expression mediated by PPARβ/δ because of the constitutively high expression of the receptor [2]. Comparison of wild-type and *Pparβ/δ*-null keratinocytes revealed that PPARβ/δ constitutively regulates expression of 482 genes. Further, activation of PPARβ/δ altered expression of 99 genes that required PPARβ/δ. The ultimate phenotype resulting from ligand activation of PPARβ/δ in keratinocytes cannot be determined based solely on the gene expression profiles obtained from these studies but they are consistent with previous studies showing that activating PPARβ/δ in keratinocytes and/or skin promotes terminal differentiation, inhibits cell proliferation, improves barrier function and inhibits inflammation [11,32-35]. Thus, it is also worth noting that activating PPARβ/δ can induce terminal differentiation and modulate lipid metabolism in keratinocytes coincident with changes in the regulation of target genes observed in these studies, consistent with previous work [32,36,37]. Expression of *Pdpk1*, *Ilk*, or *Pten* was not altered by ligand activation of PPARβ/δ in the present studies, which is also in line with a previous study [38], but in contrast to another study [39]. Increased expression of *Hb-Egf* and many pro-inflammatory genes was also reported in human keratinocytes and skin of transgenic mice over-expressing human PPARβ/δ, respectively, following activation with a PPARβ/δ ligand [40,41]. However, changes in expression of these genes were not observed in the present studies. Further work is needed to determine the reason for these inconsistencies.

Genes were categorized into eight distinct response types representing essentially every combination of transcriptional activation and/or repression in response to exogenous ligand and/or PPARβ/δ disruption. Interestingly, the type I and II responses that occur independent of exogenous ligand were the most commonly observed (79%). There are two existing models that could explain this regulation. First, PPARβ/δ can dynamically occupy chromatin in association with co-repressors leading to repression of gene expression [14]. This model explains why type I genes are induced in *Pparβ/δ*-null keratinocytes in the absence of exogenous ligand. Whether an endogenous ligand(s) is part of this complex remains uncertain. ChIP-seq analysis confirmed promoter occupancy of PPARβ/δ in some genes exhibiting a type I response. The second model is that the PPARβ/δ-RXR heterodimer in association with co-activators and RNA polymerase II is dynamically binding chromatin as a result of the presence of an endogenous ligand or ligands, and available chromatin binding sites as a result of the activities of DNA modifying enzymes [15-18]. Thus, when PPARβ/δ is disrupted, the expression of these type II genes is reduced. This is in agreement with the promoter occupancy of PPARβ/δ detected by ChIP-seq for some genes exhibiting a type II response. The fact that occupancy of PPARβ/δ was not detected for all of the type I and II genes is likely due to: 1) the dynamic fluid nature of PPARβ/δ binding with chromatin, which has multiple levels of regulation, 2) the fact that PPREs for these genes could exist in chromatin at distant sites not in close proximity to the TSS [15-18], or 3) local chromatin status at some PPREs may preclude binding at those sites [18]. Of the functional categories of genes that exhibited type I or II responses, approximately 50% of these categories were altered by both response types, suggesting common factors that induce these changes are somewhat independent of the direction of the regulation observed (e.g. repression or activation).

Type III and IV responses that were dependent on exogenous ligand activation, were the next most common type of gene regulation observed (16%). These responses were characterized by a lack of constitutive regulation by PPARβ/δ, but regulatory changes arising after treatment with exogenous ligand. Type III responses are consistent with dynamic nuclear receptor-mediated up-regulation of target gene expression [15-18]. Indeed, less promoter occupancy of PPARβ/δ was found in the absence of exogenous ligand and increased promoter occupancy of PPARβ/δ was noted following ligand activation. It is curious to note that expression of type II target genes was not increased by an exogenous ligand, since it appears that constitutive expression of type II genes is driven by an endogenous ligand(s). In contrast, constitutive expression of type III response genes did not appear to be altered by an endogenous ligand, and was only increased by an exogenous ligand. There are at least two mechanisms that could explain these differences. First, the presence of an endogenous ligand in relatively high concentration (or at sufficient concentration to saturate the receptor) that also exhibits higher affinity for PPARβ/δ as compared to GW0742 could explain why an exogenous ligand does not further increase expression of a type II response gene. This would suggest that there are different endogenous ligands that cause differential conformational changes in the receptor following ligand activation and/or differences in the recruitment of co-effector proteins to the complex to modulate specific subsets of PPARβ/δ target genes. Alternatively, the concentration of an exogenous ligand could be greater than an endogenous ligand and sufficient to activate the receptor, and/or have higher affinity for PPARβ/δ than an endogenous ligand (e.g. the endogenous ligand is in low concentration and unable to activate PPARβ/δ and/or the endogenous ligand has low affinity). This scenario also implies that the ligand could uniquely alter receptor conformation and/or recruitment of co-effector proteins to the transcriptional complex as compared to that which occurs in response to an endogenous ligand. The data from the present studies also indicate that PPARβ/δ does not influence expression of type IV genes in the absence of exogenous ligand, but that ligand activation causes recruitment of PPARβ/δ to regions near the TSS and this increase in occupancy is associated with repression of gene expression for 28 genes. Whereas repression of gene expression observed for type I response genes could be mediated by dynamic occupancy of PPARβ/δ in the absence of exogenous ligands in complex with co-repressors as suggested by other studies [14], the precise mechanism that underlies this exogenous ligand-dependent effect is uncertain and requires further studies. In general, there was a lack of overlap between the functional categories of regulatory pathways modulated by type I/II as compared to type III/IV response genes. This supports the view that PPARβ/δ is integrated into distinct transcriptional response pathways mediated by distinct mechanisms that may be influenced by more than one endogenous ligand. The fact that PPARβ/δ has a relatively large ligand binding domain as compared to other PPARs [42] and nuclear receptors supports the idea that PPARβ/δ can accommodate more than one endogenous ligand. Moreover, this analysis also suggested that PPARβ/δ can tightly bind more than one type of fatty acid, a feature that interfered with solving the crystal structure of the ligand binding domain [43], consistent with the hypothesis that more than one endogenous ligand exists for PPARβ/δ.

Mixed responses were observed for a much smaller cohort of PPARβ/δ target genes (5%). Type V response genes exhibited responses that were similar to those found with type I and III genes because in the absence of exogenous ligand and PPARβ/δ, expression was higher yet ligand activation of PPARβ/δ caused an increase in expression. Conversely, type VI response genes exhibited responses that were similar to those found with type II and IV genes because in the absence of exogenous ligand and PPARβ/δ, expression was lower and ligand activation of PPARβ/δ caused a decrease in expression. One mechanism that explains these response types is that a ligand-mediated switch occurs between repression and activation (type V) or vice-versa (type VI) [13]. For example, when PPARβ/δ is disrupted, expression of *Angptl4* is enhanced because PPARβ/δ represses expression, whereas ligand activation of PPARβ/δ increases expression of *Angptl4*. This phenomenon was inversed but also observed with type VI genes, consistent with a ligand-mediated release of an activating complex. However, it is important to point out that constitutive expression *Angptl4* and other type V response genes were typically much lower than the levels observed following activation by an exogenous ligand. This is consistent with a model where PPARβ/δ represses without an endogenous ligand and that binding of ligand to the receptor causes recruitment of transcriptional proteins and increased transcription after forming a transcriptional complex that increases transcription and/or outcompetes the repressive complex for repression. Alternatively, an exogenous ligand could alter the ratio of repressive PPARβ/δ complexes to activated PPARβ/δ complexes limiting the availability of PPARβ/δ to form repressive complexes on chromatin. Results from the analysis of type V genes in the present studies is in contrast to previous work by others showing comparable levels of expression of genes following either PPARβ/δ knockdown or activation by an exogenous ligand in human WPMY1 myofibroblast cells [13], for genes exhibiting similar regulation as observed in the present studies. This could reflect differences in: 1) the concentration of available ligand, 2) chromatin structure near the *Angptl4* gene, and/or 3) co-effector proteins recruited to alter chromatin structure. The single type VII response gene exhibited a response that was similar to that found with type II and III genes because in the absence of exogenous ligand and PPARβ/δ, expression was modestly lower and ligand activation of PPARβ/δ with GW0742 caused an increase in expression. The type VII response is consistent with a model whereby an endogenous ligand drives constitutive expression and an exogenous ligand increases this expression through mechanisms described above for type II and III response types. In contrast, type VIII response genes exhibited a response that was similar to those found with type I and IV genes because in the absence of exogenous ligand and PPARβ/δ, expression was modestly higher and ligand activation of PPARβ/δ with GW0742 caused a decrease in expression. The type VIII response is consistent with a model whereby constitutive expression is repressed and exogenous ligand also causes repression through mechanisms described above for type I and IV response types.

There are several possible explanations for the lack of detecting PPARβ/δ occupancy near the TSS of the different response type genes. It is possible that PPARβ/δ regulates these genes in regions further away than ± 10 kb from the TSS, or that regulation is mediated by another direct PPARβ/δ target gene that in turn directly regulates the target genes. This mechanism is likely for the type VII response gene because promoter occupancy of PPARβ/δ was not detected within 10 kb of the TSS. Alternatively, PPARβ/δ could occupy the regulatory regions of the response genes, but the ChIP may not be sensitive enough to effectively pull down chromatin with bound PPARβ/δ, which could be influenced by the relative ability of the antibody to bind with PPARβ/δ. The relative antibody binding to PPARβ/δ occupying chromatin could be impaired if: 1) conformational changes resulting from different co-effector molecules bound to PPARβ/δ are present, limiting access of the receptor to the antibody, 2) PPARβ/δ is indirectly bound to chromatin as part of a larger regulatory complex, 3) proteins are bound to PPARβ/δ as a result of the crosslinking step of the ChIP, and/or 4) the residence time of PPARβ/δ is too brief to register a signal in the ChIP-seq analysis because the receptor is rapidly exchanging with chromatin as observed with many other receptors [15-18].

PPARβ/δ target genes were recently identified from microarray analysis of human myofibroblast-like cells following either siRNA knockdown of PPARβ/δ or ligand activation of PPARβ/δ in these cells [13]. Adhikary and colleagues identified 595 genes that were regulated by a PPARβ/δ ligand in human WPMY1 cells [13], whereas only 130 genes were specifically regulated by ligand activation of PPARβ/δ in mouse keratinocytes in the present study (Additional file 3: Table S4). Of the 130 genes that were regulated by ligand activation of PPARβ/δ in mouse keratinocytes, 24 (19%, Additional file 4: Table S5) were also regulated by ligand activation of PPARβ/δ in human WPMY1 cells [13]. Adhikary and colleagues also identified 3704 genes that were regulated following knockdown of PPARβ/δ in human WPMY1 cells [13]. In contrast, 482 genes were differentially regulated in the absence of PPARβ/δ expression in mouse keratinocytes in the present study. Of the 482 genes that were differentially regulated by PPARβ/δ in mouse keratinocytes as detected by comparing wild-type and *Pparβ/δ*-null cells, 79 (16%, Additional file 4: Table S5) were also regulated by disrupting expression of PPARβ/δ in human WPMY1 cells [13]. Thus, of the 612 genes that were regulated by PPARβ/δ in mouse primary keratinocytes, 103 of these genes were also regulated by PPARβ/δ in WPMY1 cells (Additional file 4: Table S5). For approximately 50% of these genes, the response type exhibited was identical between mouse primary keratinocytes and WPMY1 cells (Additional file 4: Table S5). Many interchanges of response types were observed between the remaining genes but interchanges from type II to type I, type III to type I, and type I to type II were slightly more commonly noted in mouse primary keratinocytes as compared to WPMY1 cell (Additional file 4: Table S5). Collectively, these observations demonstrate that PPARβ/δ regulates some common sets of genes in human and mouse cells, but that there can also be differences in the molecular targets and the types of regulation observed. These differences might be due to the presence or absence of one or more endogenous ligand, differences in accessibility to regulatory regions of chromatin, species differences in the sequences of binding motifs, differences in the approach used to delete/knockdown PPARβ/δ (e.g. genetic versus siRNA), and/or possible species differences in the expression levels of the three PPARs between the two cell types.

The present studies identified eight different, PPARβ/δ-dependent response types of genes in mouse keratinocytes. In contrast, others characterized only three different PPARβ/δ-dependent response types in human WPMY1 cells [13]. To facilitate comparisons with the present study, data from Adhikary [13] was re-examined and indeed, all eight different response types were evident in these data. In mouse keratinocytes, constitutive expression of 482 (79%) target genes was regulated by PPARβ/δ through type I and II responses. In human WPMY1 cells, 2728 genes (84%) were also regulated through either a type I or type II response [13]. Type III and IV responses observed following activation of PPARβ/δ with an exogenous ligand were observed for 99 genes (16%) in mouse keratinocytes while 345 target genes (11%) were regulated by similar mechanisms in human WPMY1 cells [13]. Lastly, types V-VIII responses were observed for 31 genes (11%) in mouse keratinocytes, whereas 186 genes (6%) were modulated similarly in WPMY1 cells [13]. These data suggest that regulation of gene expression in mouse keratinocytes and human WPMY1 cells is likely mediated in large part by the one or more endogenous ligand, and that activation of PPARβ/δ with an exogenous ligand modulates expression of a relatively smaller set of genes as compared to those that are regulated by PPARβ/δ endogenously.

ChIP-seq analysis revealed new insight into the functional role of PPARβ/δ in the regulation of gene expression in keratinocytes. PPARβ/δ is constitutively enriched on chromatin on chromosomes 7, 9, 11 and 17, and in response to exogenous ligand activation, PPARβ/δ is enriched on chromatin located on chromosomes 2, 4, 7 and 11. These findings indicate an important role for PPARβ/δ in regulating genes encoded on these chromosomes. Interestingly, while ChIP-seq demonstrated that PPARβ/δ was present near ~6700 genes in the mouse genome, only 203 of these genes were regulated by PPARβ/δ when compared with microarray analysis. This is likely due in part to the presence of PPARβ/δ in intronic sequences that may or may not be functional. One mechanism that might explain the occupancy of PPARβ/δ on chromatin not associated with genes that were found to be regulated based on microarray analysis is that PPARβ/δ may require the presence of other transcription factors or signaling molecules in order to modulate gene expression. For example, the aryl hydrocarbon receptor (AHR) occupies the interleukin 6 (*IL6*) promoter but does not modulate expression of IL6 unless NF-kB becomes activated through IL1β-dependent signaling [44]. This type of signaling paradigm has not been examined to date for PPARβ/δ, and results from the present studies suggest this possibility could exist and should be examined in future studies. This suggests that while 612 genes were regulated specifically by PPARβ/δ, many of these changes appear to be mediated by mechanisms that are secondary to effects induced by the direct target genes. Alternatively, it remains possible that there are more direct target genes, but the regulatory elements are more distal than ± 10 kb from TSS.

ChIP-seq analysis provided a unique opportunity to begin to examine the molecular mechanisms by which PPARβ/δ differentially regulates gene expression. A consensus PPRE was derived from these analyses and was comparable to PPREs identified for other PPARβ/δ target genes [13,29]. No consistent differences in the PPRE sequences were identified that were able to distinguish between the different response types. However, the presence of binding site motifs for other transcription factors was observed that distinguished between effects observed with and without exogenous ligand. For example, the ETS binding sites were commonly present near the PPRE of genes that were modulated in the presence or absence of exogenous ligand, whereas the CREB/ATF/AP1 binding sites were commonly present near the PPRE of genes that were modulated only in the presence of exogenous ligand. Interestingly, different patterns of consensus binding sites of various transcription factors were also noted near the PPRE of genes that exhibited types I, II and III responses, but not for the other five response types. Of particular interest, is the novel finding that PPARβ/δ cooperates with ATF4 in modulating expression of some target genes. This suggests that PPARβ/δ requires cooperation with other transcription factors to specifically regulate subsets of genes. A similar phenomenon has also been found for other transcription factors [45-47]. While the physiological role of ATF4 in modulating PPARβ/δ-dependent gene expression and function requires further investigation, this type of interaction might begin to explain some of the complex regulation associated with the dynamic and fluid nature of nuclear receptor binding with chromatin.

## Conclusions

Keratinocytes express high levels of PPARβ/δ and thus provide an ideal model to study the role of this transcription factor in gene expression. Comparisons of gene expression profiles between wild-type and *Pparβ/δ*-null keratinocytes treated with and without a highly specific ligand identified 612 genes that were modulated either constitutively or by the addition of an exogenous ligand. Many of these changes in gene expression appear to be modulated by the presence of an endogenous ligand because expression is altered when PPARβ/δ is genetically silenced. ChIP-seq analysis revealed that only 203 of these 612 genes exhibit direct binding of PPARβ/δ within ± 10 Kb of the TSS. This suggests that: 1) PPARβ/δ may indirectly regulate some of the 612 genes, 2) the dynamic nature of PPARβ/δ binding and interacting with chromatin prevents detection of receptor occupancy by ChIP-seq, and/or 3) PPARβ/δ may directly regulate these genes by binding with chromatin at more distant sites. The diversity in response types detected from this analysis supports a model of PPARβ/δ interacting with chromatin through dynamic binding and interactions; differences in receptor activity induced by endogenous versus exogenous ligands may explain part of this diversity. The presence of additional transcription factors can influence the activity of PPARβ/δ in keratinocytes. Interestingly, there can be considerable overlap in target genes and response types observed between human and mouse cells.

## Methods

### Chemicals

GW0742 was kindly provided by Drs. Andrew Billin and Timothy Willson.

### Isolation and culture of mouse keratinocytes

Animal experiments were approved by the Institutional Animal Care and Use Committee at The Pennsylvania State University, which conforms to the Guide for the Care and Use of Laboratory Animals published by the National Institutes of Health. Keratinocytes were isolated from newborn mouse skin from wild-type or *Pparβ/δ*-null mice [35], and cultured as previously described [32,48]. Keratinocytes were cultured until ~80% confluent and then treated with 1 μM GW0742 or vehicle control (dimethylsulfoxide) for up to twenty-four hours and then used to isolated chromatin or RNA.

### Microarray analysis

Total RNA was isolated from wild-type or *Pparβ/δ*-null keratinocytes, treated with or without GW0742 for twenty-four hours, using TRIZOL reagent (Invitrogen, Carlsbad, CA) and purified with an RNeasy Mini Kit (Qiagen, Valencia, CA). One hundred nanograms of total RNA per sample was prepared for analysis with the GeneChip Mouse Gene 1.0 ST Array (Affymetrix, Santa Clara, CA) according to the manufacturer’s instructions. The Robust Multichip Average (RMA) approach was used for normalization of microarray data using the R/Bioconductor package as previously described [49]. To identify genes that were significantly regulated by PPARβ/δ or GW0742, a false discovery rate (FDR) cut-off of 0.25 and a fold-change of 1.3 were used. The DAVID algorithm was used to functionally categorize genes involved in different biological process as previously described [27]. Data have been deposited in NCBI’s Gene Expression Omnibus (GEO) database (http://www.ncbi.nlm.nih.gov/geo) and are accessible through GEO Series accession number GSE32498.

### Quantitative realtime polymerase chain reaction (qPCR)

Total RNA was isolated from wild-type or *Pparβ/δ*-null keratinocytes, treated with or without GW0742 for twenty-four hours as described above. qPCR was performed as previously described [50] using different primer sets (Additional file 5: Table S1).

### Chromatin immunoprecipitation (ChIP)

The ChIP-IT Express kit (Active Motif, Carlsbad, CA) was used to isolate chromatin for ChIP. Mouse primary keratinocytes were treated with or without 0.2 μM GW0742 for four hours. Cells were then treated with 1% formaldehyde for 10 min followed by glycine stop solution (125 mM) for 5 minutes. Cells were washed twice with ice-cold phosphate buffered saline, collected by scraping, and then centrifuged for 10 minutes at 2,500 rpm at 4°C. Cells were then snap frozen and stored at −80°C. Frozen cell pellets were resuspended in ice-cold lysis buffer (50 mM Tris–HCl, pH 8, 1% SDS, 10 mM EDTA, and protease inhibitor cocktail) and incubated at 4°C for 30 minutes, then homogenized with 10 strokes in a Dounce homogenizer. Nuclei were collected by centrifugation for 10 minutes at 2,500 rpm at 4°C then resuspended in ice-cold shearing buffer and incubated on ice for 10 minutes. Shearing was performed by sonication using 30-second pulses in a Diagenode Bioruptor (Diagenode, Sparta, NJ) to produce fragments ranging in size from 300-500 bp. Forty micrograms of DNA was used per immunoprecipitation (IP), or four micrograms per 10% input. Each IP was performed overnight at 4°C in a reaction mixture comprising sheared chromatin, protein G magnetic beads, and either: 1) an anti-acetylated histone 4 (Upstate Biotechnology, Lake Placid, NY); 2) an anti-PPARβ/δ antibody 8099 [2], an anti-ATF4 (Santa Cruz Biotechnology, Santa Cruz, CA) or 4) rabbit or goat IgG (Santa Cruz Biotechnology, Santa Cruz, CA). Following centrifugation, washing, and elution, the samples were reverse cross-linked by overnight incubation at 65°C and treated with proteinase K. Quantitative polymerase chain reactions (qPCR) were performed to amplify an intronic PPRE region of the *Angptl4* gene or the *Adrp* gene as previously described [20,51]. To validate putative binding regions proximal to target genes, ChIP assays were performed as described above using primers designed to amplify regions encoding between 75 and 300 bp (Additional file 5: Table S1).

### ChIP-seq

ChIP-seq was performed twice with the same samples to obtain between 15,000,000 and 23,000,00 sequence reads of ~36 base pairs per sample using the Illumina Genome Analyzer at the National Cancer Institute, Center for Cancer Research Sequencing Facility. The sequence reads were quality filtered and mapped to the mouse genome (*Mus musculus*, mm9 assembly, [52]) using Bowtie [53] with the following settings (−n 2 –e 70 –m −1-k 1). Aligned results from both sequencing runs were combined.

### Peak calling

Regions of local enrichment of 36-mer short-read tags were identified using a peak calling algorithm essentially as described previously [54]. Input samples were normalized to match the number of tags in the corresponding ChIP data. Enrichment of tags within a 150 bp target window after subtraction of normalized input tags was gauged relative to a 200 kb background window using a binomial distribution model. Each tag window was assigned a probability p given by the ratio (# of uniquely mappable base pairs in the 150 bp window)/(# of uniquely mappable base pairs for the 200 kb window). The overall z-score for the observed number of tags in the target window was defined by z = (n – μ)/σ, where σ was the standard deviation, μ was the expected number of tags overlapping the target window, with n and N observed tags overlapping the target window and background window, respectively. A putative peak was defined as a 150 bp window whose peak z-score was >60. Peaks overlapping by >1 bp with non-specific peaks identified in knockout ChIP samples were excluded. Finally, the filtered peaks for wild-type ± GW0742 were mapped to their nearest transcriptional start sites and intersected with differentially regulated genes identified from expression profiling (see below) to yield the final target gene sets.

### Transcriptional profiling and response type determination

Probe names were mapped to gene names using the manufacturer’s provided annotations for the Affymetrix Mouse Gene 1.0 ST Array. Differentially regulated genes were categorized into eight response types according to patterns of repression or induction by ligand and/or receptor deletion using a fold-change threshold of ± 1.3 and an FDR cutoff of 0.25.

### Identification of DNA binding motifs

To identify consensus peroxisome proliferator response element (PPRE) motifs for PPARβ/δ in the binding region set, the loci associated with each peak loci were expanded to yield 400 bp regions for motif searching. A list of 53 validated PPRE consensus sequences obtained from the literature [29] were used to produce a TRANSFAC matrix representing the known DR1 PPRE motif [30]. The ‘Screen Motif’ tool in the Cistrome platform was used to identify sequences within the 400 bp peak regions matching this consensus motif, or the half site (M01282) [55]. The ‘SeqPos’ tool was used to identify additional motifs statistically enriched within the 400 bp peak regions by searching the curated cistrome motif database encompassing the TRANSFAC, JASPAR, uniPROBE, and hPDI motif databases [56]. The same analyses were performed on 400 bp peak regions identified in *Pparβ/δ*-null samples, and motifs enriched in both wild-type and *Pparβ/δ*-null sample regions were omitted to correct for background. Related motifs were identified using the Phylogeny Inference Package (PHYLIP) via STAMP [30,57], and the Newick-format tree plotted using MEGA [58].

### Functional category enrichment

Gene ontology analysis was performed using DAVID to identify the PANTHER functional gene categories enriched within the eight response types using the official gene names as identifiers [27,59,60]. A *P* value cutoff of 0.05 was applied after the Benjamini-Hochberg procedure for correction of multiple hypothesis testing [27,59].

### ATF4 knockdown and characterization of gene expression

Knockdown of ATF4 in SP1 cells was achieved by infecting cells using the manufacturer’s recommended protocol with lentiviral shRNA vectors (Sigma-Aldrich, St. Louis. MO) encoding the following four sequences: 1) *Atf4* shRNA1: 5′–CCGG**GCGAGTGTAAGGAGCTAGAAA**CTCGAG**TTTCTAGCTCCTTACATTCGC**TTTTTG–3′; 2) *Atf4* shRNA2: 5′–CCGG**CCAGAGCATTCCTTTAGTTTA**CTCGAG**TAAACTAAAGGAATGCTCTGG**TTTTTG–3′; 3) *Atf4* shRNA3: 5′–CCGG**CCTCTAGTCCAAGAGACTAAT**CTCGAG**ATTAGTCTCTTGGACTAGAGG**TTTTTG–3′; 4) control shRNA 5′–CCGG**CAACAAGATGAAGAGCACCAA**CTCGAG**TTGGTGCTCTTCATCTTGTTG**TTTTT–3′. Cells were infected with vectors for 48 hours before selection with puromycin for another 48 hours. Puromycin-resistant SP1 cells were subcultured and treated with or without GW0742 (1 μM) for 24 hours, before isolation of mRNA or protein. qPCR analysis and quantitative western blots were performed as previously described [50].

## Competing interests

The authors declare that they have no competing interests.

## Authors’ contributions

CK carried out ChIP assays, knockdown studies, bioinformatic analysis and drafted the manuscript. MB carried out ChIP assays, participated in the microarray analysis and helped draft the manuscript. BZ carried out knockdown studies, performed qPCR analysis, and participated in the microarray analysis. SB and SJ carried out bioinformatic analysis of the ChIP-seq data. GH, YS and FG participated in the design and coordination of the studies, interpretation of ChIP-seq experiments and helped draft the manuscript. JP conceived of the study, participated in the design and coordination of the studies, and oversaw the drafting of the manuscript. All authors read and approved the final manuscript.

## Supplementary Material

Additional file 1 **Table S2. **612 PPARβ/δ-dependent genes sorted by response type. Click here for file

Additional file 2 **Table S3.** 203 direct PPARβ/δ target genes sorted by response type. Click here for file

Additional file 3 ** Table S4.** 130 genes modulated by ligand activation of PPARβ/δ in mouse primary keratinocytes. Click here for file

Additional file 4 ** Table S5. **103 PPARβ/δ-dependent genes common between Khozoie et al. and Adhikary et al. Click here for file

Additional file 5 **Table S1. **Primers used for the study. Click here for file
