# Supplementary material for: Analysis of the peroxisome proliferator-activated receptor-β/δ (PPARβ/δ) cistrome reveals novel co-regulatory role of ATF4
Source: BMC Genomics. 2012 Nov 24;13:665. doi: 10.1186/1471-2164-13-665 (PMC3556323; doi:10.1186/1471-2164-13-665)
Supplement: Additional file 1 — Table S2. 612 PPARβ/δ-dependent genes sorted by response type. [file 1471-2164-13-665-S1.pdf]

Supplemental Table 2: Khozoie et al
612 PPARb/d-dependent genes sorted by response type

RefSeq_ID	Gene_Symbol	type
NM_007642	Cd28	1
NM_198028	Serpinb10	1
NM_009063	Rgs5	1
NM_173388	Slc43a2	1
NM_011657	Tulp3	1
NM_172653	Slc39a10	1
NM_007381	Acadl	1
NM_021295	Lancl1	1
NM_001037999	Dbi	1
NM_053095	Il24	1
NM_009227	Snrpe	1
NM_213616	Atp2b4	1
NM_001134741	Tdrd5	1
NM_009062	Rgs4	1
NM_001002927	Penk	1
NM_001161822	Rgs17	1
NM_009538	Plagl1	1
NM_001159544	Frk	1
NM_001081208	Hs3st5	1
NM_011565	Tead2	1
DQ459435	Gm4924	1
NM_146239	Pctk2	1
NM_001002268	Gpr126	1
NM_173437	Nav1	1
NM_013508	Elk3	1
NM_017372	Lyz2	1
NR_003517	Pisd-ps1	1
NM_146015	Efemp1	1
NM_027917	Shroom1	1
NM_029537	Tmem98	1
NM_171824	Pgbd5	1
NM_133769	Cyfip2	1
NM_001161355	Timd2	1
BC055827	Olfr1372-ps1	1
NM_013472	Anxa6	1
BC069874	2810408A11Rik	1
NM_009139	Ccl6	1
NM_008238	Foxn1	1
NM_008542	Smad6	1
NM_147219	Abca5	1
XR_034692	Gm9229	1
XR_033945	Gm9282	1

XR_034974	LOC677565	1
AK046228	B230354O11Rik	1
AK132630	Gm9292	1
NM_181395	Pxdn	1
NM_178628	Atl1	1
NM_015812	Rgs6	1
NM_010496	Id2	1
NM_009234	Sox11	1
NM_153457	Rtn1	1
NM_001024474	Diras2	1
NM_029679	Fam65b	1
NM_011452	Serpinb9b	1
NM_013867	Bcar3	1
NM_010104	Edn1	1
NM_145940	Wipi1	1
NM_029847	Arsk	1
NM_012026	Rgnef	1
NM_008369	Il3ra	1
NM_011894	Sh3bp5	1
NM_172601	Rab2b	1
NM_013514	Epb4.9	1
NM_011125	Pltp	1
NM_029341	Capsl	1
NM_008530	Ly6f	1
NM_019518	Grasp	1
NM_009528	Wnt7b	1
NM_001033633	Slc2a13	1
ENSMUST000000	Gm9106	1
NM_026163	Pkp2	1
NM_178924	Upk1b	1
NM_172825	Gpr128	1
NM_183281	2310005G13Rik	1
NM_007739	Col8a1	1
NM_001011807	Olfr191	1
NM_016694	Park2	1
NM_019730	Nme3	1
NM_008804	Pde9a	1
NM_013813	Epb4.1l3	1
NM_027455	Qpct	1
NM_027457	5730437N04Rik	1
NM_009342	Dynlt1	1
NM_027644	4931440B09Rik	1
NM_011956	Nubp2	1
NM_029942	Prelid2	1
NM_011864	Papss2	1
NM_019699	Fads2	1
NM_175013	Pgm5	1
NM_001159649	Lax1	1
NM_001113569	Stxbp1	1
NM_177190	Tcp11l1	1

NM_144804	Depdc7	1
NM_026514	Cdc42ep3	1
NM_172863	Zfp697	1
XM_915925	Gm6394	1
NM_008619	Mov10	1
NM_033525	Npnt	1
NM_133871	Ifi44	1
NM_024243	Fuca1	1
NM_001008232	Asap3	1
NM_010244	Fv1	1
NM_178406	Gpr153	1
BC137640	AI464131	1
NM_007670	Cdkn2b	1
NM_023476	Tinagl1	1
NM_001083119	Ptpru	1
XM_620107	Gm13213	1
NM_011122	Plod1	1
NM_011929	Clcn6	1
NM_021604	Agrn	1
NM_011348	Sema3e	1
NR_027137	Gm10565	1
NM_001112744	Arhgef16	1
NR_003522	Abhd1	1
NM_001001980	Limch1	1
NR_003966	Atp10d	1
NM_198702	Lphn3	1
NM_018760	Slc4a4	1
BC006604	9130213B05Rik	1
NM_010203	Fgf5	1
BC049770	1700007G11Rik	1
NM_026486	Tctn2	1
NM_146257	Slc29a4	1
NM_133903	Spon2	1
NM_019494	Cxcl11	1
NM_207683	Pik3c2g	1
NM_023580	Epha1	1
NM_009527	Wnt7a	1
NM_145937	Sumf1	1
NM_033648	Fxyd4	1
NM_020008	Clec7a	1
NM_138648	Olr1	1
NM_013546	Hebp1	1
NM_007486	Arhgdib	1
NM_007948	Ercc1	1
NM_013705	Zfp30	1
NM_001033131	Krtdap	1
BC112410	2210412E05Rik	1
NM_020583	Isg20	1
NM_011035	Pak1	1
NM_022879	Myl7	1

NM_181820	Tmc4	1
NM_026111	Qpctl	1
NM_177036	Ceacam19	1
NM_027514	Pvr	1
NM_178384	Zfp74	1
NM_153679	Cpt1c	1
NM_027903	Dhdh	1
NM_001039472	Kif21b	1
NM_008594	Mfge8	1
NM_011101	Prkca	1
NM_175136	Rnf122	1
NM_172911	D8Ertd82e	1
NM_001024617	Inpp4b	1
NM_020584	Terf2ip	1
NM_021502	Trappc2l	1
NM_008430	Kcnk1	1
NM_008737	Nrp1	1
NM_013517	Fcer2a	1
NM_183315	Ctxn1	1
NM_008605	Mmp12	1
NM_019471	Mmp10	1
NM_010493	Icam1	1
NM_021339	Cdon	1
NM_023831	1500035H01Rik	1
NM_022004	Fxyd6	1
NM_007503	Fxyd2	1
NM_009034	Rbp2	1
NM_178020	Hyal3	1
BC115525	9230110C19Rik	1
NM_173777	Olfm2	1
NM_026282	Spc24	1
NM_172922	Ankk1	1
NM_009916	Ccr4	1
NM_001077361	Fhl1	1
NM_010340	Gpr50	1
NM_010941	Nsdhl	1
NM_023270	Rnf128	1
NM_178712	Gpr64	1
NM_021476	Cysltr1	1
NM_153319	Amot	1
NM_001081052	Nhs	1
NM_012011	Eif2s3y	1
	Erdr1	1
NM_018781	Egr3	2
NM_001025602	Il1rl1	2
NM_007855	Twist2	2
NM_023755	Tcfcp2l1	2
NM_178691	Yod1	2
NM_145509	5430435G22Rik	2
NM_177243	Slc26a9	2

NM_145977	Slc45a3	2
NM_022018	Fam129a	2
NM_145535	Sdcbp2	2
NM_028820	1700017B05Rik	2
NM_010518	Igfbp5	2
NM_027154	Tmbim1	2
AF064749	Col6a3	2
NM_025866	Cdca7	2
NM_008567	Mcm6	2
NM_177839	Tnn	2
NM_175686	Prrx1	2
NM_022563	Ddr2	2
NM_011825	Grem2	2
NM_001081114	Clip3	2
NM_001122893	Fyn	2
BC079613	6330442E10Rik	2
NM_177372	Dna2	2
NM_010118	Egr2	2
NM_023794	Etv5	2
NM_010512	Igf1	2
NM_001001932	Eea1	2
NM_007833	Dcn	2
NM_007792	Csrp2	2
NM_011915	Wif1	2
NM_007837	Ddit3	2
NM_008921	Prim1	2
NM_021412	Mmp19	2
NM_172308	Mthfd1l	2
NM_008481	Lama2	2
NM_007548	Prdm1	2
NM_029770	Unc5b	2
NM_001135567	1190007I07Rik	2
NM_153543	Aldh1l2	2
NM_010441	Hmga2	2
NM_009636	Aebp1	2
NM_176982	Fbxo48	2
NM_007897	Ebf1	2
NM_175643	Adamts2	2
NM_007413	Adora2b	2
NM_011786	Aloxe3	2
NM_145099	Trpv3	2
NM_013654	Ccl7	2
NM_007742	Col1a1	2
NM_053180	Ccrk	2
NM_010345	Grb10	2
NM_029327	Lyrm7	2
NM_008744	Ntn1	2
NM_011340	Serpinf1	2
NM_178309	Brip1	2
NM_009783	Cacna1g	2

NM_001013381	Rsad1	2
NM_008815	Etv4	2
NM_013614	Odc1	2
NM_023275	Rhoj	2
NM_145447	Mfsd7c	2
NM_024223	Crip2	2
NM_012038	Vsnl1	2
AK148766	3110053B16Rik	2
AK144596	G730007D18Rik	2
NM_011812	Fbln5	2
NM_026535	Serpina12	2
NM_020036	Calm4	2
NM_001008706	Calm5	2
NM_175660	Hist1h2ab	2
NM_025711	Aspn	2
BC147515	Gm906	2
NM_010049	Dhfr	2
NM_001113550	4833420G17Rik	2
NM_018859	Akr1e1	2
NR_003568	Gpr137b-ps	2
NM_022317	Slc28a3	2
NM_028186	Nkd2	2
NM_027182	Trip13	2
NM_028245	Zfp131	2
BC147211	4931406H21Rik	2
NM_134084	Ppif	2
NM_028994	Pck2	2
NM_033325	Loxl2	2
NM_145981	Phyhip	2
NM_172812	Htr2a	2
NM_026214	Kctd4	2
NM_001101433	Zcchc24	2
BC049685	2610528A11Rik	2
NM_016972	Slc7a8	2
NM_152894	Pop1	2
NM_018865	Wisp1	2
NM_008495	Lgals1	2
NM_182928	Adm2	2
NM_145218	Wbscr17	2
NM_008764	Tnfrsf11b	2
NM_027435	Atad2	2
NM_008555	Masp1	2
NM_028973	Lrrc15	2
NM_011145	Ppard	2
NM_025789	Rshl2a	2
NM_144515	Zfp52	2
BC076634	BC049807	2
NM_001025373	4930432O21Rik	2
NM_145484	Zfp758	2
NM_177822	Mslnl	2

NM_019688	Rapgef4	2
NM_011896	Spry1	2
NM_001008424	Cdsn	2
NM_001099632	Rnf39	2
NM_147155	Tagap1	2
NM_011581	Thbs2	2
NM_023824	Paqr4	2
NM_172935	Amdhd2	2
NM_025718	Dnase1l2	2
NM_022880	Slc29a1	2
NM_176922	Itga11	2
NM_001146268	Pdgfrb	2
NM_133237	Apcdd1	2
NM_013685	Tcf4	2
NM_011412	Slit3	2
NM_145492	Zfp521	2
NM_178347	Cdc23	2
NM_011898	Spry4	2
BC038020	Fam111a	2
NM_029993	Mlana	2
NM_175459	Glis3	2
NM_011701	Vim	2
NM_019498	Olfm1	2
NM_207298	Cercam	2
NM_001146350	Eng	2
NM_009398	Tnfaip6	2
NM_175512	Dhrs9	2
NM_021398	Slc43a3	2
NM_011234	Rad51	2
NM_026929	Chac1	2
NM_175034	Slc24a5	2
NM_001001309	Itga8	2
BC117777	2310002J15Rik	2
NM_011576	Tfpi	2
NM_145526	P2rx3	2
NM_011957	Creb3l1	2
NM_201367	Gpr176	2
NM_007993	Fbn1	2
NM_175093	Trib3	2
NM_001099327	100043387	2
NM_008875	Pld1	2
NR_027966	Zmat3	2
NM_019393	Exosc9	2
NM_008006	Fgf2	2
NM_183221	Fat4	2
NM_015784	Postn	2
NM_207263	Pglyrp4	2
NM_007802	Ctsk	2
NM_009323	Tbx15	2
NM_026993	Ddah1	2

NM_023644	Mccc1	2
NM_027810	Bbs7	2
NM_001040695	Uevld	2
NM_025984	Lce1a1	2
NM_026822	Lce1b	2
NM_028625	Lce1a2	2
NM_027137	Lce1d	2
NM_026394	Lce1f	2
NM_029667	Lce1i	2
XM_001478521	Gm7055	2
NM_133859	Olfml3	2
NM_031186	Ndst3	2
NM_145953	Cth	2
NM_001111027	Runx1t1	2
NM_021362	Pappa	2
NM_008546	Mfap2	2
NM_008726	Nppb	2
NM_009400	Tnfrsf18	2
AK137636	9930005F22Rik	2
NM_022814	Svep1	2
BC066147	2310067E19Rik	2
NM_010329	Pdpn	2
NM_001025365	Miip	2
NM_173401	Fbxo44	2
NM_001081557	Camta1	2
NM_011985	Mmp23	2
NM_028977	Lrrc17	2
NM_025796	Mrpl33	2
NM_011058	Pdgfra	2
NM_001122733	Kit	2
NM_011027	P2rx7	2
NM_001042421	Kntc1	2
NM_007743	Col1a2	2
NM_022890	Cldn12	2
NM_026770	Cgref1	2
NM_146007	Col6a2	2
NM_146162	Tmem119	2
NM_022017	Trpv4	2
NM_175092	Rhof	2
NM_008871	Serpine1	2
NM_008788	Pcolce	2
NM_032000	Trps1	2
BC151018	A430107O13Rik	2
NM_001081306	Ptprz1	2
NM_027926	Cpa4	2
BC047154	A930038C07Rik	2
NM_007992	Fbln2	2
NM_010585	Itpr1	2
NM_199033	Tsen2	2
NM_172271	Slc6a17	2

NM_026218	Fgfr1op2	2
NM_172309	Arntl2	2
NM_026221	Ppfibp1	2
NM_001130190	Sgce	2
NM_012055	Asns	2
NM_026637	Ggct	2
NM_026004	Nt5c3	2
NM_008638	Mthfd2	2
NM_009610	Actg2	2
NM_010751	Mxd1	2
NM_010924	Nnmt	2
NM_008157	Gpr19	2
BC141220	2310014L17Rik	2
NM_008627	Meis3	2
NM_026938	Tmem160	2
NM_009201	Slc1a5	2
NM_007949	Ercc2	2
NM_022409	Zfp296	2
NM_009999	Cyp2b10	2
NM_133712	Klk10	2
NM_028660	Klk9	2
NM_008940	Klk8	2
NM_011872	Klk7	2
NM_016849	Irf3	2
NM_009737	Bcat2	2
NM_001146049	Htatip2	2
NM_001040085	Sytl2	2
NM_011858	Odz4	2
NM_027629	Pgm2l1	2
NM_019391	Lsp1	2
NM_007469	Apoc1	2
NM_009696	Apoe	2
NM_019940	Zfp111	2
NM_020262	Zfp109	2
NM_001039185	Ceacam1	2
NM_001037707	Zfp27	2
NM_009884	Cebpg	2
NM_011131	Pold1	2
NM_030693	Atf5	2
NM_026555	Rcn3	2
NM_018876	Fut2	2
NM_010129	Emp3	2
NM_001005247	Hps5	2
NM_001013368	E2f8	2
NM_030728	9930013L23Rik	2
NM_177382	Cyp2r1	2
NM_013742	Cars	2
NM_007631	Ccnd1	2
NM_019728	Defb4	2
NM_054044	Gpr124	2

NM_153581	Gpm6a	2
NM_022331	Herpud1	2
NM_019733	Rbpms	2
NM_001005863	Mtus1	2
NM_010332	Ednra	2
NM_011925	Cd97	2
NM_021491	Smpd3	2
NM_011404	Slc7a5	2
NM_019552	Abcb10	2
NM_133733	9030425E11Rik	2
NM_009382	Thy1	2
NM_023061	Mcam	2
NM_001083587	Tns3	2
NM_001013373	Tmprss13	2
NM_008360	Il18	2
NM_013496	Crabp1	2
NM_011352	Sema7a	2
NM_009445	Ttk	2
NM_033314	Slco2a1	2
NM_173781	Rab6b	2
NM_053159	Mrpl3	2
NM_025651	Aste1	2
NM_029631	Abhd14b	2
NM_153168	Lars2	2
NM_007691	Chek1	2
NM_008775	Pafah1b2	2
NM_025848	Sdhd	2
NM_023483	1110032A03Rik	2
NM_010729	Loxl1	2
NM_001081242	Tln2	2
NM_001110271	Abhd14a	2
NM_001044384	Timp1	2
NM_016886	Gria3	2
NM_019926	Mtm1	2
NM_007542	Bgn	2
NM_175271	Lpar4	2
NM_026838	Srpx2	2
NM_010797	Mid1	2
NM_008892	Pola1	2
	Sts	2
NM_010827	Msc	3
NM_009949	Cpt2	3
NM_001081206	Dgki	3
NM_053090	Fam126a	3
NM_007981	Acsl1	3
NM_010234	Fos	3
NM_009434	Phlda2	3
NM_001033217	Prickle1	3
NM_011198	Ptgs2	3
NM_013914	Snai3	3

NM_019993	Aldh9a1	3
NM_027950	Osgin1	3
NM_008869	Pla2g4a	3
NM_010476	Hsd17b7	3
NM_146006	Lss	3
NM_001031772	Lin28b	3
NM_177368	Tmtc2	3
NM_017366	Acadvl	3
NM_025638	Gdpd1	3
NM_012006	Acot1	3
NM_009747	Bdkrb2	3
NM_053155	Clmn	3
NM_011868	Peci	3
NM_008046	Fst	3
NM_025341	Abhd6	3
NM_177814	Erc2	3
NM_008125	Gjb2	3
NM_207708	Syngr1	3
NM_018790	Arc	3
NM_011638	Tfrc	3
NM_053261	Impa2	3
NM_177470	Acaa2	3
NM_010235	Fosl1	3
XR_034011	Gm5246	3
NM_138595	Gldc	3
NM_145533	Smox	3
NM_001024145	Pla2g4f	3
NM_008256	Hmgcs2	3
NM_013609	Ngf	3
NM_009196	Slc16a1	3
NM_130450	Elovl6	3
NM_009626	Adh7	3
NM_025794	Etfdh	3
NM_016966	Phgdh	3
NM_029522	Gpsm2	3
NM_172687	Coq3	3
NM_080289	Grhpr	3
NM_024255	Hsdl2	3
NM_026172	Decr1	3
NM_007408	Plin2	3
NM_172383	Tmem125	3
NM_153526	Insig1	3
NM_145558	Hadhb	3
NM_015756	Shroom3	3
NM_178878	Hadha	3
NM_001102414	Slc2a9	3
NM_145839	Rasgef1b	3
NM_023516	2310016C08Rik	3
NM_009908	Cmas	3
NM_013743	Pdk4	3

NM_026695	Etfb	3
NM_011671	Ucp2	3
NM_009627	Adm	3
NM_001130479	Nucb2	3
NM_172759	Ces5	3
BC027185	2210023G05Rik	3
NM_026931	1810011O10Rik	3
NM_025436	Sc4mol	3
NM_008317	Hyal1	3
NM_019750	Nat6	3
NM_178753	Spin4	3
NM_198108	Morn4	4
NM_201644	Ugt1a9	4
NM_009369	Tgfbi	4
NM_001081175	Itpkb	4
NM_016917	Slc40a1	4
NM_172411	2310007B03Rik	4
NM_146240	Rassf9	4
NM_027890	Susd2	4
NM_173733	Suox	4
NM_008885	Pmp22	4
NM_001005341	Ypel2	4
NM_153782	Fam20a	4
NM_024188	Oxct1	4
NM_029436	Klhl24	4
NM_144855	Cbs	4
BC096371	D18Ertd653e	4
NM_001033280	Gm94	4
BC120879	4833423E24Rik	4
NM_025416	Them5	4
NM_178786	Skint4	4
NM_011402	Slc34a2	4
NM_025826	Acadsb	4
NM_178704	Dpy19l3	4
NM_027998	Cldn23	4
NM_001025577	Maf	4
NM_178415	Bbs9	4
NM_026674	Aph1c	4
NM_016913	Porcn	4
NM_020581	Angptl4	5
NM_053178	Acsbg1	5
NM_028817	Acsl3	5
NM_011704	Vnn1	5
NM_009760	Bnip3	5
NM_027906	1300010F03Rik	5
NM_013495	Cpt1a	5
NM_009127	Scd1	5
NM_008009	Fgfbp1	5
NM_145130	Lpcat3	5
NM_001004153	AU018091	5

NM_020520	Slc25a20	5
NM_027884	Tns1	6
NM_007695	Chi3l1	6
NM_010401	Hal	6
NM_027997	Serpina9	6
NM_133357	Krt75	6
NM_020622	Fam3b	6
NM_001039042	Klk13	6
NM_001145960	Slc37a2	6
NM_011139	Pou2f3	6
NM_011333	Ccl2	7
NM_027406	Aldh1l1	8
NM_146017	Gabrp	8
NM_011430	Sncg	8
NM_009155	Sepp1	8
NM_172671	Lgr4	8
NM_025285	Stmn2	8
NM_030601	Clca2	8
NM_172752	Sorbs2	8
NM_017369	Gabre	8
