# Supplementary material for: Analysis of the peroxisome proliferator-activated receptor-β/δ (PPARβ/δ) cistrome reveals novel co-regulatory role of ATF4
Source: BMC Genomics. 2012 Nov 24;13:665. doi: 10.1186/1471-2164-13-665 (PMC3556323; doi:10.1186/1471-2164-13-665)
Supplement: Additional file 2 — Table S3. 203 direct PPARβ/δ target genes sorted by response type. [file 1471-2164-13-665-S2.pdf]

Supplemental Table 3; Khozoie et al
203 Direct PPARb/d target genes, sorted by response type

RefSeq_ID	Gene_Symbol	type
NR_003522	Abhd1	1
NM_021604	Agrn	1
BC137640	AI464131	1
NM_013472	Anxa6	1
NM_007486	Arhgdib	1
NM_001112744	Arhgef16	1
NM_029847	Arsk	1
NM_213616	Atp2b4	1
NM_026514	Cdc42ep3	1
NM_172911	D8Ertd82e	1
NM_001037999	Dbi	1
NM_144804	Depdc7	1
NM_027903	Dhdh	1
NM_001024474	Diras2	1
NM_013813	Epb4.1l3	1
NM_019699	Fads2	1
NM_029679	Fam65b	1
NM_001077361	Fhl1	1
NM_008238	Foxn1	1
NR_027137	Gm10565	1
NM_178406	Gpr153	1
NM_010340	Gpr50	1
NM_013546	Hebp1	1
NM_178020	Hyal3	1
NM_010493	Icam1	1
NM_053095	Il24	1
NM_008430	Kcnk1	1
NM_001039472	Kif21b	1
NM_001033131	Krtdap	1
NM_021295	Lancl1	1
NM_001159649	Lax1	1
NM_017372	Lyz2	1
NM_008594	Mfge8	1
NM_022879	Myl7	1
NM_173437	Nav1	1
NM_033525	Npnt	1
NM_008737	Nrp1	1
NM_001002927	Penk	1
NM_009538	Plagl1	1
NM_011122	Plod1	1
NM_011125	Pltp	1
NM_011101	Prkca	1
NM_001083119	Ptpru	1
NM_027455	Qpct	1

NM_001161822	Rgs17	1
NM_153457	Rtn1	1
NM_011894	Sh3bp5	1
NM_173388	Slc43a2	1
NM_018760	Slc4a4	1
NM_011565	Tead2	1
NM_023476	Tinagl1	1
NM_021502	Trappc2l	1
NM_011657	Tulp3	1
NM_178924	Upk1b	1
NM_145940	Wipi1	1
NM_013705	Zfp30	1
NM_178384	Zfp74	1
NM_028820	1700017B05Rik	2
BC117777	2310002J15Rik	2
BC141220	2310014L17Rik	2
BC147211	4931406H21Rik	2
NM_001110271	Abhd14a	2
NM_029631	Abhd14b	2
NM_175643	Adamts2	2
NM_182928	Adm2	2
NM_007413	Adora2b	2
NM_009636	Aebp1	2
NM_011786	Aloxe3	2
NM_172309	Arntl2	2
NM_027435	Atad2	2
BC076634	BC049807	2
NM_178309	Brip1	2
NM_009783	Cacna1g	2
NM_001008706	Calm5	2
NM_013742	Cars	2
NM_007631	Ccnd1	2
NM_011925	Cd97	2
NM_178347	Cdc23	2
NM_001039185	Ceacam1	2
NM_026770	Cgref1	2
NM_026929	Chac1	2
NM_001081114	Clip3	2
NM_007742	Col1a1	2
NM_146007	Col6a2	2
NM_013496	Crabp1	2
NM_024223	Crip2	2
NM_007792	Csrp2	2
NM_009999	Cyp2b10	2
NM_007837	Ddit3	2
NM_177372	Dna2	2
NM_025718	Dnase1l2	2
NM_001013368	E2f8	2
NM_007949	Ercc2	2
NM_183221	Fat4	2

NM_011812	Fbln5	2
NM_026637	Ggct	2
NM_054044	Gpr124	2
NM_010345	Grb10	2
NM_022331	Herpud1	2
NM_001146049	Htatip2	2
NM_172812	Htr2a	2
NM_016849	Irf3	2
NM_176922	Itga11	2
NM_010585	Itpr1	2
NM_001122733	Kit	2
NM_028973	Lrrc15	2
NM_028977	Lrrc17	2
NM_019391	Lsp1	2
NM_008555	Masp1	2
NM_023061	Mcam	2
NM_008627	Meis3	2
NM_008546	Mfap2	2
NM_145447	Mfsd7c	2
NM_011985	Mmp23	2
NM_177822	Mslnl	2
NM_001005863	Mtus1	2
NM_008726	Nppb	2
NM_026004	Nt5c3	2
NM_133859	Olfml3	2
NM_028994	Pck2	2
NM_008788	Pcolce	2
NM_011058	Pdgfra	2
NM_008892	Pola1	2
NM_011131	Pold1	2
NM_152894	Pop1	2
NM_015784	Postn	2
NM_011145	Ppard	2
NM_001013381	Rsad1	2
NM_145535	Sdcbp2	2
NM_025848	Sdhd	2
NM_177243	Slc26a9	2
NM_022317	Slc28a3	2
NM_022880	Slc29a1	2
NM_145977	Slc45a3	2
NM_022814	Svep1	2
NM_023755	Tcfcp2l1	2
NM_011581	Thbs2	2
NM_009382	Thy1	2
NM_001081242	Tln2	2
NM_001013373	Tmprss13	2
NM_009400	Tnfrsf18	2
NM_175093	Trib3	2
NM_027182	Trip13	2
NM_199033	Tsen2	2

NM_001040695	Uevld	2
NM_029770	Unc5b	2
NM_012038	Vsnl1	2
NM_178691	Yod1	2
NM_022409	Zfp296	2
NM_145484	Zfp758	2
BC027185	2210023G05Rik	3
NM_025341	Abhd6	3
NM_009626	Adh7	3
NM_019993	Aldh9a1	3
NM_009747	Bdkrb2	3
NM_172759	Ces5	3
NM_130450	Elovl6	3
NM_026695	Etfb	3
NM_053090	Fam126a	3
NM_010234	Fos	3
NM_008046	Fst	3
NM_178878	Hadha	3
NM_145558	Hadhb	3
NM_008256	Hmgcs2	3
NM_010476	Hsd17b7	3
NM_024255	Hsdl2	3
NM_001031772	Lin28b	3
NM_013609	Ngf	3
NM_027950	Osgin1	3
NM_016966	Phgdh	3
NM_009434	Phlda2	3
NM_008869	Pla2g4a	3
NM_007408	Plin2	3
NM_001033217	Prickle1	3
NM_025436	Sc4mol	3
NM_015756	Shroom3	3
NM_001102414	Slc2a9	3
NM_145533	Smox	3
NM_013914	Snai3	3
NM_207708	Syngr1	3
NM_172383	Tmem125	3
NM_172411	2310007B03Rik	4
NM_026674	Aph1c	4
NM_178415	Bbs9	4
NM_144855	Cbs	4
NM_178704	Dpy19l3	4
NM_153782	Fam20a	4
NM_001081175	Itpkb	4
NM_198108	Morn4	4
NM_024188	Oxct1	4
NM_008885	Pmp22	4
NM_009369	Tgfbi	4
NM_025416	Them5	4
NM_053178	Acsbg1	5

NM_020581	Angptl4	5
NM_008009	Fgfbp1	5
NM_145130	Lpcat3	5
NM_020622	Fam3b	6
NM_010401	Hal	6
NM_133357	Krt75	6
NM_027406	Aldh1l1	8
NM_146017	Gabrp	8
NM_172671	Lgr4	8
