# Supplementary material for: Analysis of the peroxisome proliferator-activated receptor-β/δ (PPARβ/δ) cistrome reveals novel co-regulatory role of ATF4
Source: BMC Genomics. 2012 Nov 24;13:665. doi: 10.1186/1471-2164-13-665 (PMC3556323; doi:10.1186/1471-2164-13-665)
Supplement: Additional file 3 — Table S4. 130 genes modulated by ligand activation of PPARβ/δ in mouse primary keratinocytes. [file 1471-2164-13-665-S3.pdf]

Supplemental Table 4; Khozoie et al
130 modulated by ligand activation of PPARb/d in mouse keratinocytes

RefSeq_ID	Gene_Symbol	type
NM_010827	Msc	3
NM_009949	Cpt2	3
NM_001081206	Dgki	3
NM_053090	Fam126a	3
NM_007981	Acsl1	3
NM_010234	Fos	3
NM_009434	Phlda2	3
NM_001033217	Prickle1	3
NM_011198	Ptgs2	3
NM_013914	Snai3	3
NM_019993	Aldh9a1	3
NM_027950	Osgin1	3
NM_008869	Pla2g4a	3
NM_010476	Hsd17b7	3
NM_146006	Lss	3
NM_001031772	Lin28b	3
NM_177368	Tmtc2	3
NM_017366	Acadvl	3
NM_025638	Gdpd1	3
NM_012006	Acot1	3
NM_009747	Bdkrb2	3
NM_053155	Clmn	3
NM_011868	Peci	3
NM_008046	Fst	3
NM_025341	Abhd6	3
NM_177814	Erc2	3
NM_008125	Gjb2	3
NM_207708	Syngr1	3
NM_018790	Arc	3
NM_011638	Tfrc	3
NM_053261	Impa2	3
NM_177470	Acaa2	3
NM_010235	Fosl1	3
XR_034011	Gm5246	3
NM_138595	Gldc	3
NM_145533	Smox	3
NM_001024145	Pla2g4f	3
NM_008256	Hmgcs2	3
NM_013609	Ngf	3

NM_009196	Slc16a1	3
NM_130450	Elovl6	3
NM_009626	Adh7	3
NM_025794	Etfdh	3
NM_016966	Phgdh	3
NM_029522	Gpsm2	3
NM_172687	Coq3	3
NM_080289	Grhpr	3
NM_024255	Hsdl2	3
NM_026172	Decr1	3
NM_007408	Plin2	3
NM_172383	Tmem125	3
NM_153526	Insig1	3
NM_145558	Hadhb	3
NM_015756	Shroom3	3
NM_178878	Hadha	3
NM_001102414	Slc2a9	3
NM_145839	Rasgef1b	3
NM_023516	2310016C08Rik	3
NM_009908	Cmas	3
NM_013743	Pdk4	3
NM_026695	Etfb	3
NM_011671	Ucp2	3
NM_009627	Adm	3
NM_001130479	Nucb2	3
NM_172759	Ces5	3
BC027185	2210023G05Rik	3
NM_026931	1810011O10Rik	3
NM_025436	Sc4mol	3
NM_008317	Hyal1	3
NM_019750	Nat6	3
NM_178753	Spin4	3
NM_198108	Morn4	4
NM_201644	Ugt1a9	4
NM_009369	Tgfbi	4
NM_001081175	Itpkb	4
NM_016917	Slc40a1	4
NM_172411	2310007B03Rik	4
NM_146240	Rassf9	4
NM_027890	Susd2	4
NM_173733	Suox	4
NM_008885	Pmp22	4
NM_001005341	Ypel2	4
NM_153782	Fam20a	4
NM_024188	Oxct1	4
NM_029436	Klhl24	4
NM_144855	Cbs	4
BC096371	D18Ertd653e	4
NM_001033280	Gm94	4
BC120879	4833423E24Rik	4

NM_025416	Them5	4
NM_178786	Skint4	4
NM_011402	Slc34a2	4
NM_025826	Acadsb	4
NM_178704	Dpy19l3	4
NM_027998	Cldn23	4
NM_001025577	Maf	4
NM_178415	Bbs9	4
NM_026674	Aph1c	4
NM_016913	Porcn	4
NM_020581	Angptl4	5
NM_053178	Acsbg1	5
NM_028817	Acsl3	5
NM_011704	Vnn1	5
NM_009760	Bnip3	5
NM_027906	1300010F03Rik	5
NM_013495	Cpt1a	5
NM_009127	Scd1	5
NM_008009	Fgfbp1	5
NM_145130	Lpcat3	5
NM_001004153	AU018091	5
NM_020520	Slc25a20	5
NM_027884	Tns1	6
NM_007695	Chi3l1	6
NM_010401	Hal	6
NM_027997	Serpina9	6
NM_133357	Krt75	6
NM_020622	Fam3b	6
NM_001039042	Klk13	6
NM_001145960	Slc37a2	6
NM_011139	Pou2f3	6
NM_011333	Ccl2	7
NM_027406	Aldh1l1	8
NM_146017	Gabrp	8
NM_011430	Sncg	8
NM_009155	Sepp1	8
NM_172671	Lgr4	8
NM_025285	Stmn2	8
NM_030601	Clca2	8
NM_172752	Sorbs2	8
NM_017369	Gabre	8
