# Supplementary material for: Analysis of the peroxisome proliferator-activated receptor-β/δ (PPARβ/δ) cistrome reveals novel co-regulatory role of ATF4
Source: BMC Genomics. 2012 Nov 24;13:665. doi: 10.1186/1471-2164-13-665 (PMC3556323; doi:10.1186/1471-2164-13-665)
Supplement: Additional file 4 — Table S5. 103 PPARβ/δ-dependent genes common between Khozoie et al. and Adhikary et al. [file 1471-2164-13-665-S4.pdf]

Supplemental Table 5: Khozoie et al

103 PPARb/d-dependent genes common between Khozoie et al and Adhikary et al

RefSeq_ID	Gene_Symbol	type	Adhikary et al Type	Human_Gene	humanEntrez	mouseEntrez
NM_147219	Abca5	1	2	ABCA5	23461	217265
NM_021604	Agrn	1	2	AGRN	375790	11603
NM_013472	Anxa6	1	1	ANXA6	309	11749
NM_007486	Arhgdib	1	1	ARHGDIB	397	11857
NM_213616	Atp2b4	1	1	ATP2B4	493	381290
NM_013867	Bcar3	1	1	BCAR3	8412	29815
NM_007670	Cdkn2b	1	1	CDKN2B	1030	12579
NM_007739	Col8a1	1	7	COL8A1	1295	12837
NM_146015	Efemp1	1	2	EFEMP1	2202	216616
NM_024243	Fuca1	1	1	FUCA1	2517	71665
NM_001002268	Gpr126	1	6	GPR126	57211	215798
NM_019518	Grasp	1	5	GRASP	160622	56149
NM_178020	Hyal3	1	1	HYAL3	8372	109685
NM_010496	Id2	1	1	ID2	3398	15902
NM_011101	Prkca	1	3	PRKCA	5578	18750
NM_027455	Qpct	1	1	QPCT	25797	70536
NM_009062	Rgs4	1	2	RGS4	5999	19736
NM_027917	Shroom1	1	4	SHROOM1	134549	71774
NM_172653	Slc39a10	1	2	SLC39A10	57181	227059
NM_021502	Trappc2l	1	1	TRAPPC2L	51693	59005
NM_011657	Tulp3	1	2	TULP3	7289	22158
NM_030728	9930013L23Rik	2	2	KIAA1199	57214	80982
BC151018	A430107O13Rik	2	4	C7ORF58	79974	214642
NM_029631	Abhd14b	2	1	ABHD14B	84836	76491
NM_172309	Arntl2	2	8	ARNTL2	56938	272322
NM_012055	Asns	2	2	ASNS	440	27053
NM_025651	Aste1	2	3	ASTE1	28990	66595
NM_178309	Brip1	2	1	BRIP1	83990	237911
NM_001081557	Camta1	2	2	CAMTA1	23261	100072
NM_013742	Cars	2	3	CARS	833	27267
NM_011925	Cd97	2	1	CD97	976	26364
NM_178347	Cdc23	2	1	CDC23	8697	52563
NM_026770	Cgref1	2	1	CGREF1	10669	68567
NM_022890	Cldn12	2	1	CLDN12	9069	64945
NM_007742	Col1a1	2	2	COL1A1	1277	12842
NM_007743	Col1a2	2	3	COL1A2	1278	12843
NM_024223	Crip2	2	2	CRIP2	1397	68337
NM_007792	Csrp2	2	2	CSRP2	1466	13008
NM_009999	Cyp2b10	2	3	CYP2B6	1555	13088
NM_007833	Dcn	2	2	DCN	1634	13179

NM_001013368	E2f8	2	2	E2F8	79733	108961
NM_001001932	Eea1	2	1	EEA1	8411	216238
NM_023794	Etv5	2	6	ETV5	2119	104156
NM_183221	Fat4	2	7	FAT4	79633	329628
NM_007992	Fbln2	2	2	FBLN2	2199	14115
NM_008006	Fgf2	2	7	FGF2	2247	14173
NM_175459	Glis3	2	2	GLIS3	169792	226075
NM_054044	Gpr124	2	2	GPR124	25960	78560
NM_175660	Hist1h2ab	2	2	HIST1H2AE	8335	319170
NM_010441	Hmga2	2	1	HMGA2	8091	15364
NM_008360	Il18	2	2	IL18	3606	16173
NM_001122733	Kit	2	2	KIT	3815	16590
NM_010729	Loxl1	2	2	LOXL1	4016	16949
NM_172308	Mthfd1l	2	1	MTHFD1L	25902	270685
NM_010751	Mxd1	2	2	MXD1	4084	17119
NM_010924	Nnmt	2	2	NNMT	4837	18113
NM_028994	Pck2	2	1	PCK2	5106	74551
NM_008788	Pcolce	2	2	PCOLCE	5118	18542
NM_001146268	Pdgfrb	2	2	PDGFRB	5159	18596
NM_027629	Pgm2l1	2	1	PGM2L1	283209	70974
NM_152894	Pop1	2	1	POP1	10940	67724
NM_015784	Postn	2	2	POSTN	10631	50706
NM_011145	Ppard	2	2	PPARD	5467	19015
NM_026221	Ppfibp1	2	1	PPFIBP1	8496	67533
NM_008921	Prim1	2	1	PRIM1	5557	19075
NM_001013381	Rsad1	2	2	RSAD1	55316	237926
NM_011352	Sema7a	2	1	SEMA7A	8482	20361
NM_008871	Serpine1	2	2	SERPINE1	5054	18787
NM_011340	Serpinf1	2	2	SERPINF1	5176	20317
NM_021398	Slc43a3	2	2	SLC43A3	29015	58207
NM_011404	Slc7a5	2	2	SLC7A5	8140	20539
NM_011412	Slit3	2	2	SLIT3	6586	20564
NM_001040085	Sytl2	2	2	SYTL2	54843	83671
NM_013685	Tcf4	2	2	TCF4	6925	21413
NM_001044384	Timp1	2	2	TIMP1	7076	21857
NM_027154	Tmbim1	2	2	TMBIM1	64114	69660
NM_008764	Tnfrsf11b	2	2	TNFRSF11B	4982	18383
NM_027182	Trip13	2	3	TRIP13	9319	69716
NM_029770	Unc5b	2	2	UNC5B	219699	107449
NM_177470	Acaa2	3	7	ACAA2	10449	52538
NM_009627	Adm	3	2	ADM	133	11535
NM_009747	Bdkrb2	3	1	BDKRB2	624	12062
NM_009949	Cpt2	3	1	CPT2	1376	12896
NM_026172	Decr1	3	1	DECR1	1666	67460
NM_026695	Etfb	3	1	ETFB	2109	110826
NM_025794	Etfdh	3	1	ETFDH	2110	66841
NM_008046	Fst	3	2	FST	10468	14313
NM_029522	Gpsm2	3	1	GPSM2	29899	76123
NM_024255	Hsdl2	3	1	HSDL2	84263	72479
NM_053261	Impa2	3	5	IMPA2	3613	114663
NM_153526	Insig1	3	1	INSIG1	3638	231070
NM_146006	Lss	3	1	LSS	4047	16987

NM_016966	Phgdh	3	2	PHGDH	26227	236539
NM_007408	Plin2	3	5	PLIN2	123	11520
NM_178753	Spin4	3	2	SPIN4	139886	270624
NM_198108	Morn4	4	1	MORN4	118812	226123
NM_016913	Porcn	4	2	PORCN	64840	53627
NM_020581	Angptl4	5	5	ANGPTL4	51129	57875
NM_009760	Bnip3	5	2	BNIP3	664	100042570
NM_013495	Cpt1a	5	1	CPT1A	1374	12894
NM_009127	Scd1	5	1	SCD	6319	20249
NM_020520	Slc25a20	5	3	SLC25A20	788	57279
NM_172671	Lgr4	8	2	LGR4	55366	107515
