# Supplementary material for: Analysis of the peroxisome proliferator-activated receptor-β/δ (PPARβ/δ) cistrome reveals novel co-regulatory role of ATF4
Source: BMC Genomics. 2012 Nov 24;13:665. doi: 10.1186/1471-2164-13-665 (PMC3556323; doi:10.1186/1471-2164-13-665)
Supplement: Additional file 5 — Table S1. Primers used for the study. [file 1471-2164-13-665-S5.pdf]

Supplemental Table 1. Khozoie et al
Primers used for the study

**Gene Name**	**Primer Use**	**Forward Primer**	**Reverse Primer**
ABHD1	qPCR	CTTAGCTCTTGGCGTTGCTC	CAGTGTTGTTCCAGGAAGGC
ADRP	qPCR	CCCGTATTTGAGATCCGTGT	CAATTTGTGGCTCCAGCTTC
ANGPTL4	qPCR	TTCTCGCCTACCAGAGAAGTTGGG	CATCCACAGCACCTACAACAGCAC
ATF4	qPCR	GTCCCTCCAACAACAGCAAG	CTATACCCAACAGGGCATCC
CBS	qPCR	CGGACTCCCCACATTATCAC	TGTTGATTCTGACCATAGGGG
CES5	qPCR	GATGGGACCTCACATCCAAA	CATAGAGATGGGAGGCAGGA
DHDH	qPCR	CAGAAGTTCGGGAGATGGTT	AAACTTCCCTCAGAGCCTCC
HDHB	qPCR	CAGGAAGTGAAAACAAGCAATG	AGCCATGGTGACAGTGTGAG
HTATIP2	qPCR	TGATGTTGGATTCTGTTGCC	CAGCTCTGCAGACTTGAGCA
MCAM	qPCR	CCAAACTGGTGTGCGTCTT	GTGGGTACTGGCTGCTTTTC
MORN4	qPCR	TCACTTCGAGAATGGGCTTT	AACTTGCCCTGGGAAAACTC
OSGIN1	qPCR	GAGTGTCCAGACAACCCAGG	CAGGAGGTCATAGCTCGGTG
PMP22	qPCR	AGCCGTCCAACACTGCTACT	GAACAGGAACAGAGCCAGGA
PRICKLE1	qPCR	CCTGACGCGGAGGACCGCAG	ATCCAAGAGACAGCTGCCCACG
SDCBP2	qPCR	CAGTGGCTGGAGAAGAGGAC	CCACCTTCAGATCCTCCAGA
SNAI3	qPCR	GTGAAAACGCACTCCAGTCA	GGATCCTGCCAACTCCTTG
TGFB1	qPCR	ACCAGAGGAAGATCTGCGG	ATTTGAGAGCGGAAGAGCTG
TULP3	qPCR	CAAGCCAGACCTTCAGGAGA	CCCTCATCTTCCTCCTTGTC
ADRP	ChIP-qPCR	CTCGGGCCCATGTTACAGT	CGGTGAGTGTGCTGCTGAG
ANGPTL4	ChIP-qPCR	AAGGCACACAGGATAGGTTTGAAAG	GGTACTTCGTAGTCTCGGCTGAATG
MCAM	ChIP-qPCR	CCTGGGTTTCTGTAACTCTGAAATC	TGCTTATGTGAACAGGGAGTGTTAC
MORN4	ChIP-qPCR	GCTTGGCTTCTGGGCGACGACTGTC	CCACCTCTCTTTCAGATCTTTGGCC
OSGIN1	ChIP-qPCR	TGGGTACTTAGAGTGAGAGCCTGAC	CCCTGGTCCTTATAGACTTCTCTTC
PRICKLE1	ChIP-qPCR	TCCTGGGGATCTGAACTGAG	CAATTCCCTGAACCAAGTCG
SLC43A2	ChIP-qPCR	GCTCATCTTTATCGCCTTGG	AGGCAATAAATGTGGACCGA
SNAI3	ChIP-qPCR	GCTATGACTCACAGTTACAGGCCAG	CTGTGGCCCGCTCCATGTGCTTCTG
TGFB1	ChIP-qPCR	CTGCTCTCACTTCACCAGCA	TGACCTGTCCTATGCTCACG
TULP3	ChIP-qPCR	GGAACTGGAGTTACAGATGGTTGTG	CAACCCTACTCATTTAGCACTAGCC
